# Combined effects of basalt fiber geometrical characteristics on pavement performance of asphalt mixtures

**DOI:** 10.1371/journal.pone.0316173

**Published:** 2025-01-31

**Authors:** Heng Zhou, Mengxin Li, Guyue Jin, Mengyu Guo, Yingjun Jiang

**Affiliations:** 1 Nanyang Fangzao Expressway Co., Ltd, Nanyang, Henan, China; 2 School of Highway, Chang’an University, Xi’an, China; Shandong University of Technology, CHINA

## Abstract

Fibers have been widely adopted in asphalt mixture to improve its pavement performance. Lignin fiber and polyester fiber are the most popular two choices. Lignin fiber is derived from wood, which is not aligned with the principles of sustainable development. The production process for polyester fiber is more complex and costly, presenting both environmental and economic challenges in engineering applications. In contrast, basalt fiber is cost-effective, exhibit excellent wear resistance and impact toughness, and possess high mechanical strength. It is an ideal choice to improve pavement performance of asphalt mixtures. However, most of the existing studies focused on analyzing a single characteristic index of basalt fiber. They neglected the composite effects of geometric characteristics of basalt fiber, such as fiber diameter and length, on the pavement performance of asphalt mixtures at varying fiber contents. Therefore, taking the SMA-13 as an example, the combined effect of basalt fiber geometrical characteristics (fiber diameter, fiber length, and fiber content) on pavement performance are elucidated. Additionally, a random forest algorithm is adopted to perform a weight analysis of fiber characteristics and their correlation with pavement performance.

## Introduction

For an extended period, asphalt pavement has been extensively utilized in high-grade highways across our country due to its excellent mechanical properties. However, issues related to pavement deterioration in heavy-duty traffic, long longitudinal slopes, level crossings, toll plazas, and other specialized areas are increasingly common and severe. Despite implementing various measures, such as incorporating anti-rutting agents, using modified asphalt, or applying injected pavement techniques, these issues remain inadequately addressed. Fiber products offer significant advantages, including high mechanical strength and good compatibility with cement materials. The pavement performance of asphalt mixtures can be enhanced through reinforcement and toughening with these fibers. To promote high-quality construction of transportation infrastructure and improve the durability of asphalt pavement, the application of fibers in asphalt pavement engineering has emerged as a focal point in the field of road engineering.

Currently, the fiber products commonly used in asphalt pavement include lignin fiber and polyester fiber [1]. However, lignin fiber is derived from wood, leading to significant wood consumption, which is not aligned with the principles of sustainable development. The production process for polyester fiber is more complex and costly, presenting both environmental and economic challenges in engineering applications. In contrast, basalt fiber is a new type of mineral fiber recognized as a "green industrial raw material" due to its non-polluting nature. Compared to lignin and polyester fibers, basalt fiber and its products are cost-effective, exhibit excellent wear resistance and impact toughness, and possess high mechanical strength. Additionally, basalt fiber demonstrates good compatibility with cement materials and other advantageous properties, enhancing the performance of asphalt mixtures through reinforcement and toughening [2–4].

Abtahi et al. [5] compared the pavement performance of various fiber-reinforced asphalt mixtures and found that the overall pavement performance of basalt fiber asphalt mixtures was relatively superior. Xu et al. [6] selected three kinds of basalt fibers of length (3 mm, 6 mm, and 12 mm) and three kinds of diameter (7 μm, 16 μm, and 25 μm) with 9 different characteristic parameters were selected to test the properties of basalt fiber mixed asphalt mortar with different characteristic parameters. The results show that the fiber with a length of 6mm and a diameter of 7 mm has a better strengthening effect. Ahmad et al. [7] noted that when the content of basalt fiber in the asphalt mixture exceeds a certain threshold, the asphalt-aggregate ratio increases, thereby enhancing the anti-rutting and anti-cracking properties of the mixture. Morova et al. [8–10] discovered that asphalt pavement incorporating basalt fiber exhibits excellent Marshall stability, as well as high and low-temperature performance, water stability, and fatigue resistance. By creating Marshall specimens, Radziszewski et al. [11] concluded that the inclusion of basalt fiber can enhance the Marshall stability of the asphalt mixture and improve its resistance to permanent deformation, significantly extending its fatigue life. Pirmohammad et al. [12] investigated the effect of basalt fiber on the fracture toughness of asphalt mixtures using a semicircular bending test and found that the fracture toughness increased with higher fiber content but decreased with longer fiber lengths. Fan et al. [13] employed the Marshall test to determine the optimal content of basalt fiber in asphalt mixtures to be 0.3%. Ameri et al. [14] and Celauro et al. [15] mixed 0.3% basalt fiber based on the quality of the mixture and found that, compared to conventional asphalt mixtures, the amount of asphalt increased, rutting resistance and mechanical properties improved, and the resistance to permanent deformation of the mixture was enhanced. Xie et al. [16] used orthogonal test method to evaluate the asphalt pavement structure with 9 different basalt fiber contents, and adopted finite element modeling method to determine the optimal content of basalt fiber between different layers. Lei et al. [17] studied the reinforcement effect of fiber on rubber asphalt through tensile ductility test, optimized the optimal content of fiber, asphalt and gravel through fatigue cracking test, and verified the cracking resistance of fiber rubber gravel seal through fracture energy test. Huang et al. [18] conducted complex modulus tests on asphalt mixtures with fiber content of 0%, 0.1%, 0.2%, and 0.3%, respectively. Zhang et al. [19] had developed a new method that can effectively characterize the transient damage evolution of basalt fiber asphalt mixtures and evaluate their fracture properties.

Existing studies conducted both domestically and internationally indicates that basalt fiber can significantly enhance the performance of asphalt mixtures. However, most of these investigations focus on analyzing a single characteristic index of basalt fiber. They neglected the composite effects of geometric characteristics, such as fiber diameter and length, on the pavement performance at varying mixing amounts. Therefore, taking SMA-13 as an example, the combined effect of basalt fiber geometrical characteristics (fiber diameter, fiber length, and fiber content) on the pavement performance are elucidated. Additionally, this study also employs a random forest algorithm to conduct a weight analysis of fiber characteristics and its correlation with pavement performance.

## Materials and methods

### Materials

(1) Asphalt

The SBS modified asphalt produced by Maoming Xinlu Building Materials Technology Co., Ltd. is adopted in this study. The technical properties are presented in Table 1, respectively.

**Table 1:** Technical properties of modified asphalt.

Test index		Measured value	Standard value
25°C penetration (0.1 mm)		50	40~60
Penetration index PI		1.14	≥ 0
5°C ductility(cm)		42	≥ 20
Softening point (Global method) (°C)		80	≥ 60
135°C kinematic viscosity (Pa·s)		1.5	≤ 3.0
Solubility (trichloroethylene) (%)		99.81	≥ 99
25°C elastic recovery (%)		87	≥ 75
Rotating film aging test	Mass loss (%)	-0.67	≤ ±1.0
	Ductility (5°C, cm)	22	≥ 15
	Penetration ratio (25°C) (%)	69.9	≥ 65

(2) Coarse aggregates

Coarse aggregate utilized in this study is basalt crushed aggregates produced by Shaanxi Rongxin Mining Development Co., Ltd. The technical properties are presented in Table 2.

**Table 2:** Coarse aggregate technical indexes.

Test index	The following specifications (mm) coarse aggregate			Standard value
	13.2~16	9.5~13.2	4.75~9.5	
Apparent relative density	2.883	2.877	2.870	≥ 2.6
Needle flake particle content (%)	5.8	7.1	8.4	≤ 15
Water absorption (%)	0.48	0.86	0.51	≤ 2.0
Crush value (%)	14.2			≤ 26
Wear value (%)	16.1			≤ 28
Robustness (%)	6.7			≤ 12

(3) Fine aggregate

Fine aggregate utilized in this study is limestone machine-made sand produced by Shaanxi Rongxin Mining Development Co., Ltd. The technical properties are presented in Table 3.

**Table 3:** Technical indexes of fine aggregate.

Test index	Test value	Standard value
Apparent relative density	2.783	≥ 2.5
Robustness (%)	7.1	≤ 12
Methylene blue value (g/kg)	3.0	≤ 25
Angular (s)	37.1	≥ 30

(4) Mineral powder

Mineral powder utilized in this study is limestone mineral powder produced by Shuntongda Mining Co., Ltd., located in Baoji, Shaanxi. The technical properties are presented in Table 4.

**Table 4:** Mineral powder technical index.

Test index		Test value	Standard value
Apparent relative density		2.756	≥ 2.5
Water content (%)		0.4	≤ 1
Size range (%)	<0.6 mm	100	100
	<0.15 mm	96.1	90~100
	<0.075 mm	92.2	75~100
Hydrophilic coefficient		0.5	< 1
Plasticity index (%)		3.2	< 4
Heating stability		Good	Field record

(5) Basalt Fiber

**Fig 1:**
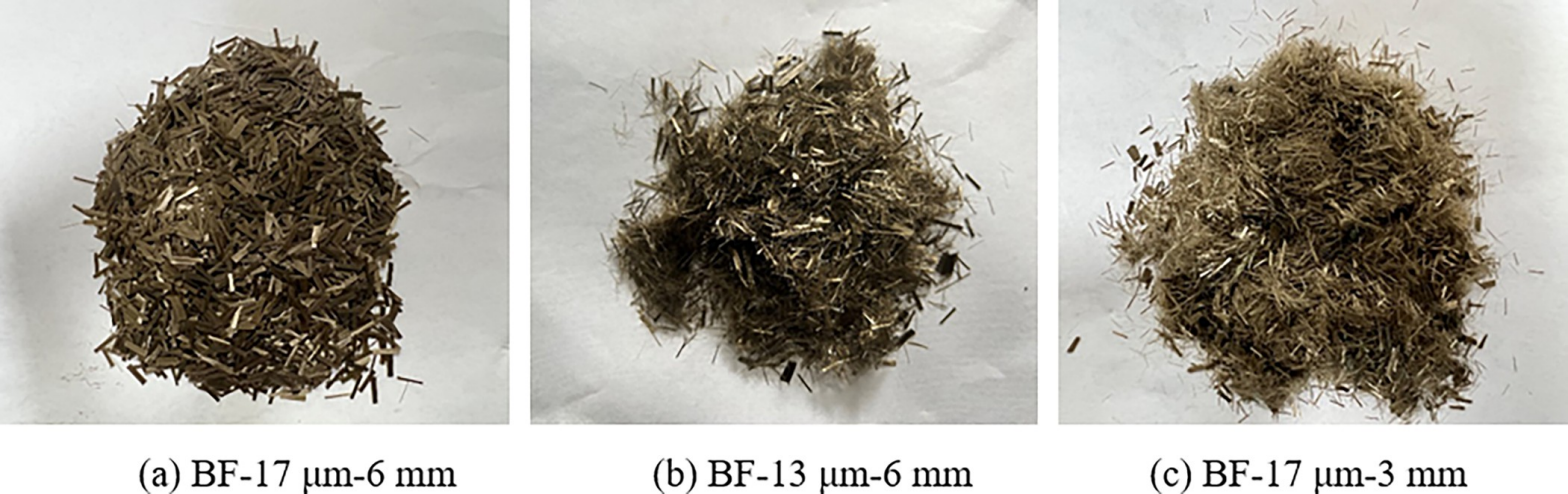
Basalt fiber.

The fundamental performance indices of basalt fiber (BF) utilized in this study are presented in Table 5, which was provided by a fiber technology company located in Jiangsu Province, as illustrated in Fig 1. The types of basalt fiber selected for this study include those with a diameter of 17 μm and a length of 6 mm, a diameter of 13 μm and a length of 6 mm, and a diameter of 17 μm and a length of 3 mm.

**Table 5:** Basic properties of basalt fiber.

Test index	Detection value		
Color	Golden-brown		
Fiber diameter (μm)	17	13	17
Length (mm)	6	6	3
Designation	BF-17 μm-6 mm	BF-13 μm-6 mm	BF-17 μm-3 mm
Density (g/cm^3^)	2.683		2.712
Melting point (°C)	1250		1250
Moisture content (%)	0.14		0.16
Breaking strength (MPa)	1570		1480
Elastic modulus (GPa)	40		80
Elongation at break (%)	2.6		3.1

(5) Gradation

The range of density gradation for the basalt fiber SMA-13 mixture is presented in Table 6. In this table, S-SMA-13 represents the dense grade of the SMA-13 mixture with a strongly integrated skeleton, while G-SMA-13 denotes the standard grade of SMA-13.

**Table 6:** Gradation.

Gradation type	Percentage of mass passing (%) through the following screen sizes (mm)									
	16	13.2	9.5	4.75	2.36	1.18	0.6	0.3	0.15	0.075
S-AMA-13	100	100	53~58	31~42	19~29	16~21	12~14	10~11	7~8	5~7
G-SMA-13	100	90~100	50~75	20~34	15~26	14~24	12~20	10~16	9~15	8~12

### Experimental methods

The rutting tests, bending tests at -10°C, and freeze-thaw splitting tests are adopted to investigate the high-temperature performance, low-temperature performance, and water stability of the asphalt mixture. All experiments are carried out according to the Chinese experimental standard [20].

## Effect of basalt fiber on optimal asphalt-aggregate ratio

Table 7 presents the optimal ratios of various basalt fiber types and their corresponding content in the SMA-13 mixture.

**Table 7:** Best asphalt-aggregate ratio of basalt fiber SMA-13.

Gradation type	Basalt fiber type	The optimum asphalt-aggregate ratio (%) using the following fiber content				
		0.2%	0.3%	0.4%	0.5%	0.6%
S-SMA-13	BF-17 μm-3 mm	5.78	5.83	5.85	5.88	5.90
	BF-17 μm-6 mm	5.79	5.83	5.86	5.89	5.91
	BF-13 μm-6 mm	5.80	5.85	5.88	5.90	5.93
G-SMA-13	BF-17 μm-3 mm	5.88	5.90	5.93	5.96	6.01
	BF-17 μm-6 mm	5.89	5.92	5.95	5.99	6.03
	BF-13 μm-6 mm	5.90	5.94	5.96	6.00	6.05

(1) Effect of fiber diameter

**Fig 2:**
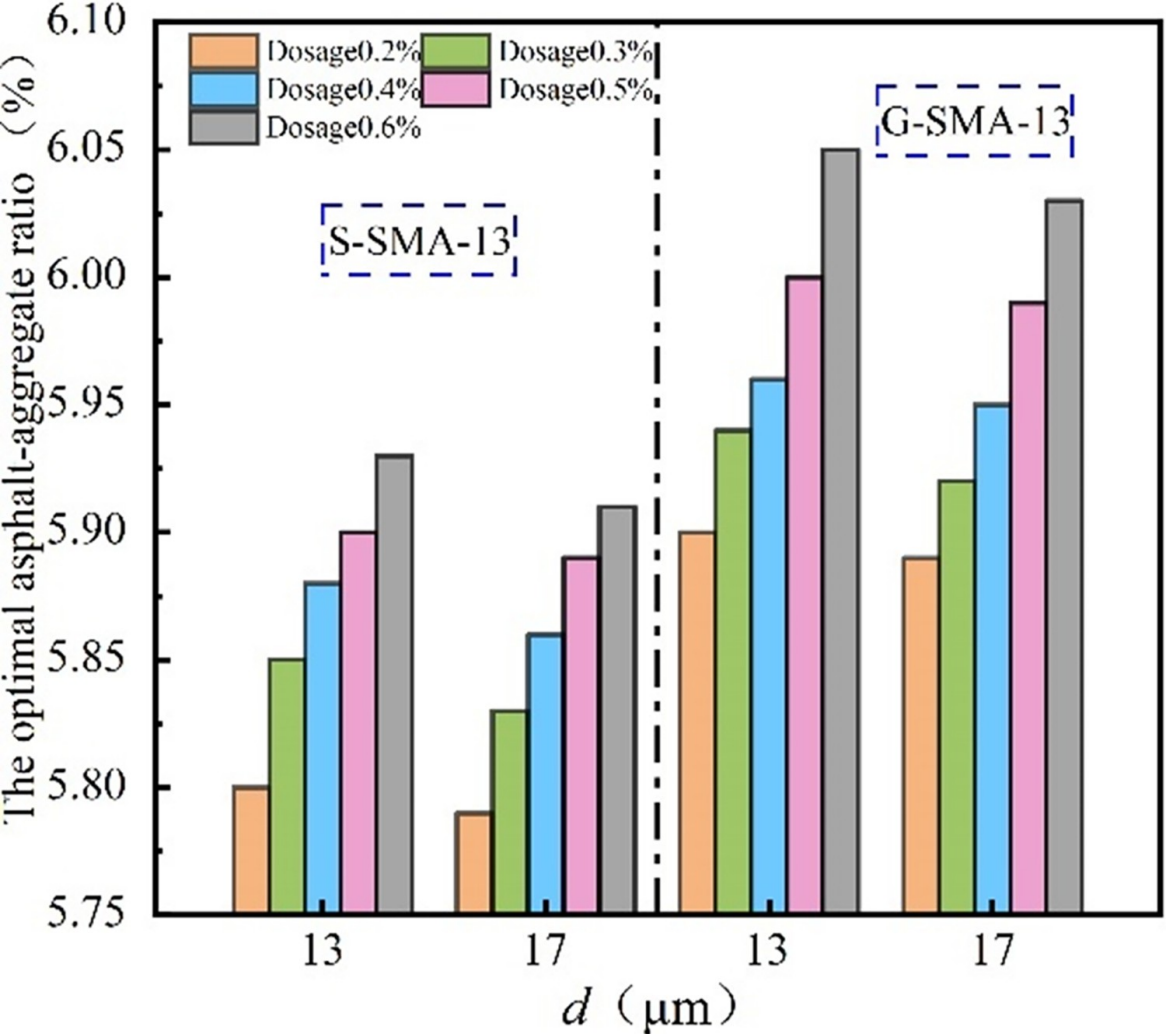
Influence rule of diameter on optimal asphalt-aggregate ratio.

The effect of basalt fiber diameter on the optimal asphalt-aggregate ratio of the SMA-13 mixture is illustrated in Fig 2.

It can be observed from Fig 2 that the optimal asphalt-aggregate ratio for SMA-13 with a basalt fiber diameter of 13 μm is greater than that for SMA-13 with a basalt fiber diameter of 17 μm. When finer basalt fibers are incorporated into the SMA-13 mixture, they possess a larger specific surface area, which not only increases the demand for adsorbed asphalt but also provides more contact points for the asphalt at the microscopic level. Consequently, the mixture appears to require a higher quantity of asphalt-aggregate ratio. Additionally, the dispersion of coarse fibers in SMA-13 is less uniform compared to that of fine fibers, resulting in a lower demand for asphalt in the mixture.

(2) Effect of fiber length

**Fig 3:**
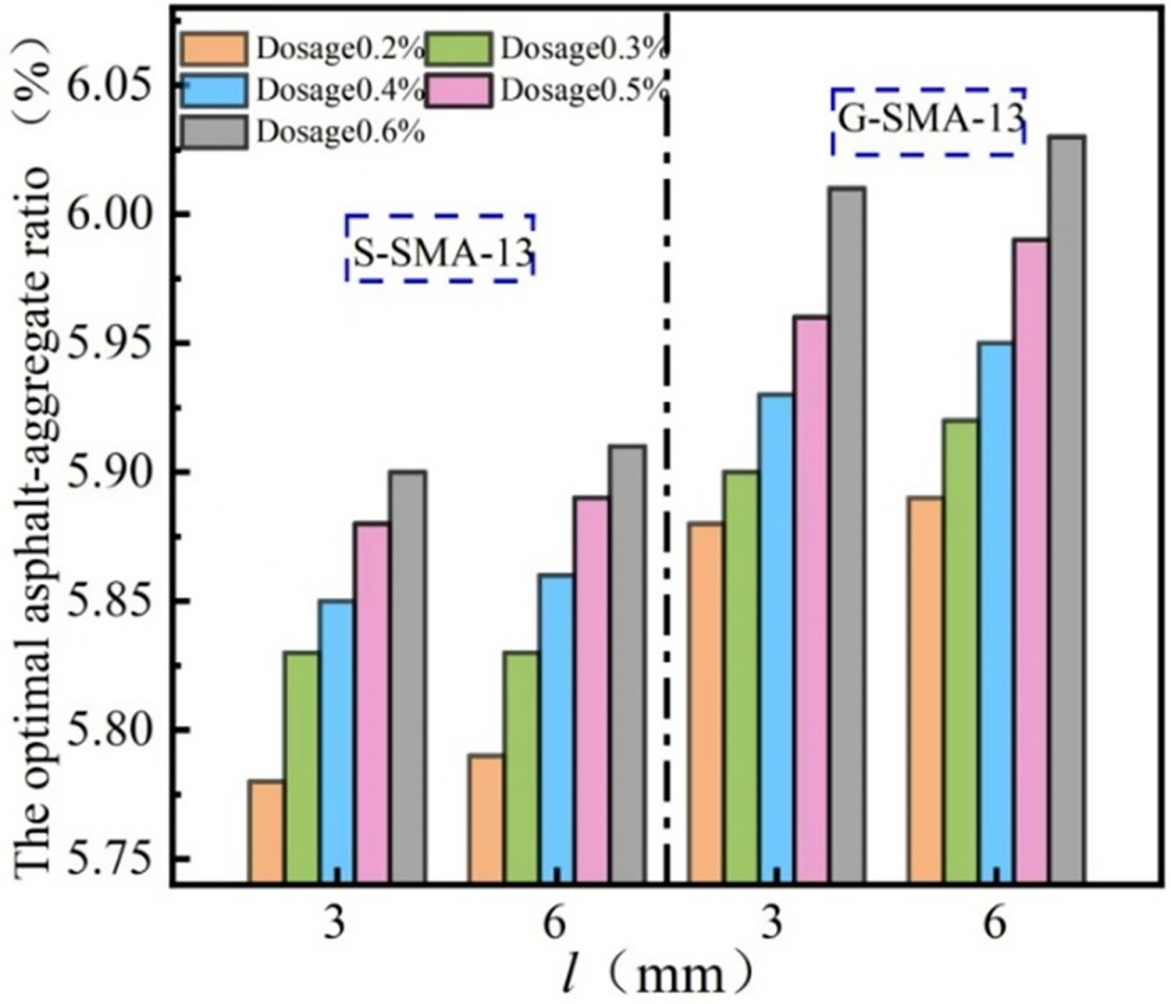
Influence of fiber length on the optimum asphalt-aggregate ratio.

The effect of basalt fiber length on the optimal asphalt-aggregate ratio of the SMA-13 mixture is illustrated in Fig 3.

It can be observed from Fig 3 that the optimal asphalt-aggregate ratio for SMA-13 with a basalt fiber length of 6 mm is greater than that for SMA-13 with a basalt fiber length of 3 mm. The incorporation of longer basalt fibers into the SMA-13 mix necessitates a greater amount of asphalt coating to effectively bridge, bond, and form a three-dimensional network structure within the mixture. Furthermore, shorter fibers are more uniformly dispersed throughout the mixture, preventing agglomeration, whereas longer fibers require additional asphalt coating in areas of local aggregation. This requirement is reflected in the increased asphalt content within the mixture.

(3) Effect of fiber content

**Fig 4:**
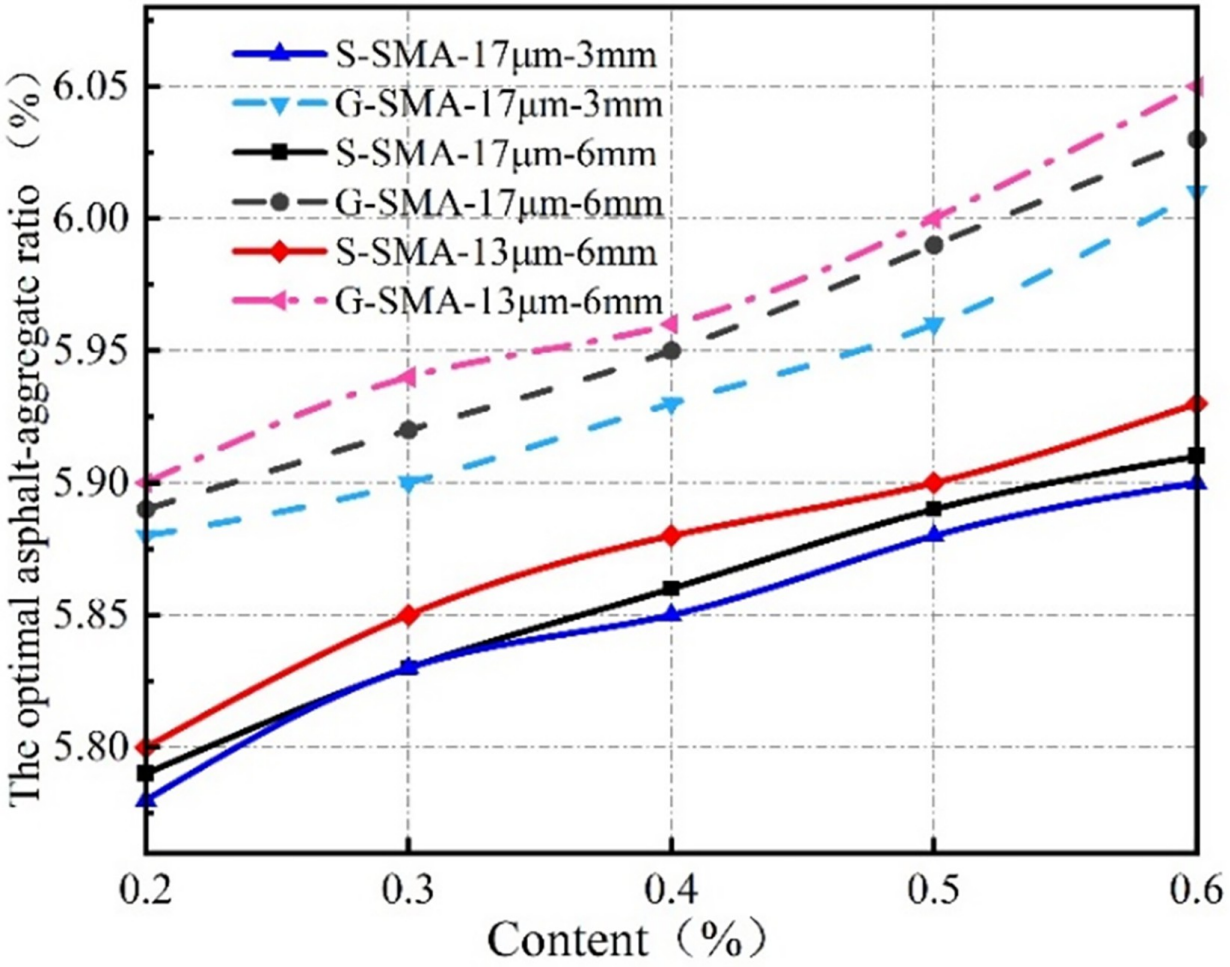
Effect of fiber content on optimal asphalt-aggregate ratio.

The effect of basalt fiber content on the optimal asphalt-aggregate ratio of the SMA-13 mixture is illustrated in Fig 4.

It can be observed from Fig 4 that the optimal asphalt-aggregate ratio of basalt fiber SMA-13 increases with the rising content of basalt fiber. This phenomenon occurs because basalt fiber possesses asphalt absorption properties, allowing it to absorb asphalt. As the fiber content increases, the total surface area of the fiber within the mixture also increases, which complicates the uniform distribution of the fiber. Consequently, a greater amount of asphalt is required to adequately cover the fiber, leading to an increase in the optimal asphalt-aggregate ratio in the mixture.

## Influence of basalt fiber on pavement performance

The influences of basalt fiber diameter, length, and content on the mechanical properties, high-temperature stability, low-temperature crack resistance, and water stability of the mixture are analyzed. This study is based on the optimal asphalt-aggregate ratio for the basalt fiber SMA-13. The optimal diameter, length, and content of the basalt fiber are determined according to the principles of maximizing mechanical and pavement performance.

### Test results

The mechanical and pavement performance test results for various types and contents of basalt fiber SMA-13 are presented in Tables 8–10. In these tables, *R*_*c*_ denotes compressive strength, RT represents splitting strength, *DS* indicates dynamic stability, RB refers to flexural tensile strength, *ε*_*B*_ signifies flexural tensile strain, *S*_*B*_ stands for bending stiffness modulus, *MS*_*0*_ denotes residual stability, and *TSR* represents the freeze-thaw splitting strength ratio.

**Table 8:** Mechanical and pavement performance of basalt fiber SMA-13 (BF-17 μm-3 mm).

Gradation type	Content (%)	*R*_c_ (MPa)	*R*_T_ (MPa)	*DS* (Times/mm)	*R*_B_ (MPa)	*ε*_B_ (10^−6^)	*S*_B_ (MPa)	*MS*_0_ (%)	*TSR* (%)
X-SMA-13	0.2	7.8	1.02	7941	10.9	2928	3731	87.7	86.7
	0.3	8.1	1.30	8552	11.3	3310	3407	88.8	88.0
	0.4	8.3	1.44	8873	11.8	3709	3174	90.5	89.0
	0.5	8.2	1.34	8253	11.5	3432	3358	89.6	88.2
	0.6	8.0	1.29	8008	11.1	3056	3624	88.5	87.2
G-SMA-13	0.2	7.4	0.94	6473	9.7	2679	3631	85.8	85.0
	0.3	7.7	1.21	6923	10.3	2864	3613	87.0	85.7
	0.4	7.8	1.27	7383	10.8	3343	3234	88.9	86.9
	0.5	7.7	1.20	7079	10.6	3043	3479	88.2	85.9
	0.6	7.6	1.16	6655	10.1	2789	3619	87.3	85.4

**Table 9:** Mechanical and pavement performance of basalt fiber SMA-13 (BF-17 μm-6 mm).

Gradation type	Content (%)	*R*_c_ (MPa)	*R*_T_ (MPa)	*DS* (Times/mm)	*R*_B_ (MPa)	*ε*_B_ (10^−6^)	*S*_B_ (MPa)	*MS*_0_ (%)	*TSR* (%)
S-SMA-13	0.2	8.0	1.08	8217	11.0	3093	3562	88.8	87.5
	0.3	8.3	1.38	8957	11.7	3492	3339	89.9	88.6
	0.4	8.5	1.51	9043	12.2	3856	3152	91.8	89.9
	0.5	8.4	1.40	8552	12.0	3688	3245	90.5	88.8
	0.6	8.3	1.32	8289	11.6	3371	3435	90.1	87.9
G-SMA-13	0.2	7.6	0.99	6923	10.2	2739	3742	86.9	85.2
	0.3	8.0	1.27	7105	10.8	3070	3528	87.8	86.0
	0.4	8.1	1.39	7714	11.2	3506	3200	89.2	87.5
	0.5	7.8	1.24	7382	10.9	3240	3379	88.4	86.7
	0.6	7.7	1.18	7052	10.7	3100	3438	87.6	85.9

**Table 10:** Mechanical and pavement performance of basalt fiber SMA-13 (BF-13 μm-6 mm).

Gradation type	Content (%)	*R*_c_ (MPa)	*R*_T_ (MPa)	*DS* (Times/mm)	*R*_B_ (MPa)	*ε*_B_ (10^−6^)	*S*_B_ (MPa)	*MS*_0_ (%)	*TSR* (%)
S-SMA-13	0.2	8.23	1.09	8400	11.6	3331	3489	89.0	88.2
	0.3	8.53	1.47	9265	12.6	4057	3105	92.7	90.4
	0.4	8.72	1.58	9087	12.4	3735	3307	91.6	89.6
	0.5	8.60	1.41	8750	12.0	3527	3404	90.4	88.9
	0.6	8.40	1.29	8591	11.8	3352	3528	89.4	88.1
G-SMA-13	0.2	7.84	1.04	7052	10.7	3044	3525	87.8	85.7
	0.3	8.12	1.34	7941	11.6	3669	3156	91.0	88.0
	0.4	8.24	1.49	7530	11.3	3478	3255	89.5	87.2
	0.5	8.07	1.39	7426	11.1	3348	3326	88.6	86.4
	0.6	7.91	1.23	7214	10.9	3221	3372	87.8	86.0

### Effect of fiber diameter

(1) Mechanical property

**Fig 5:**
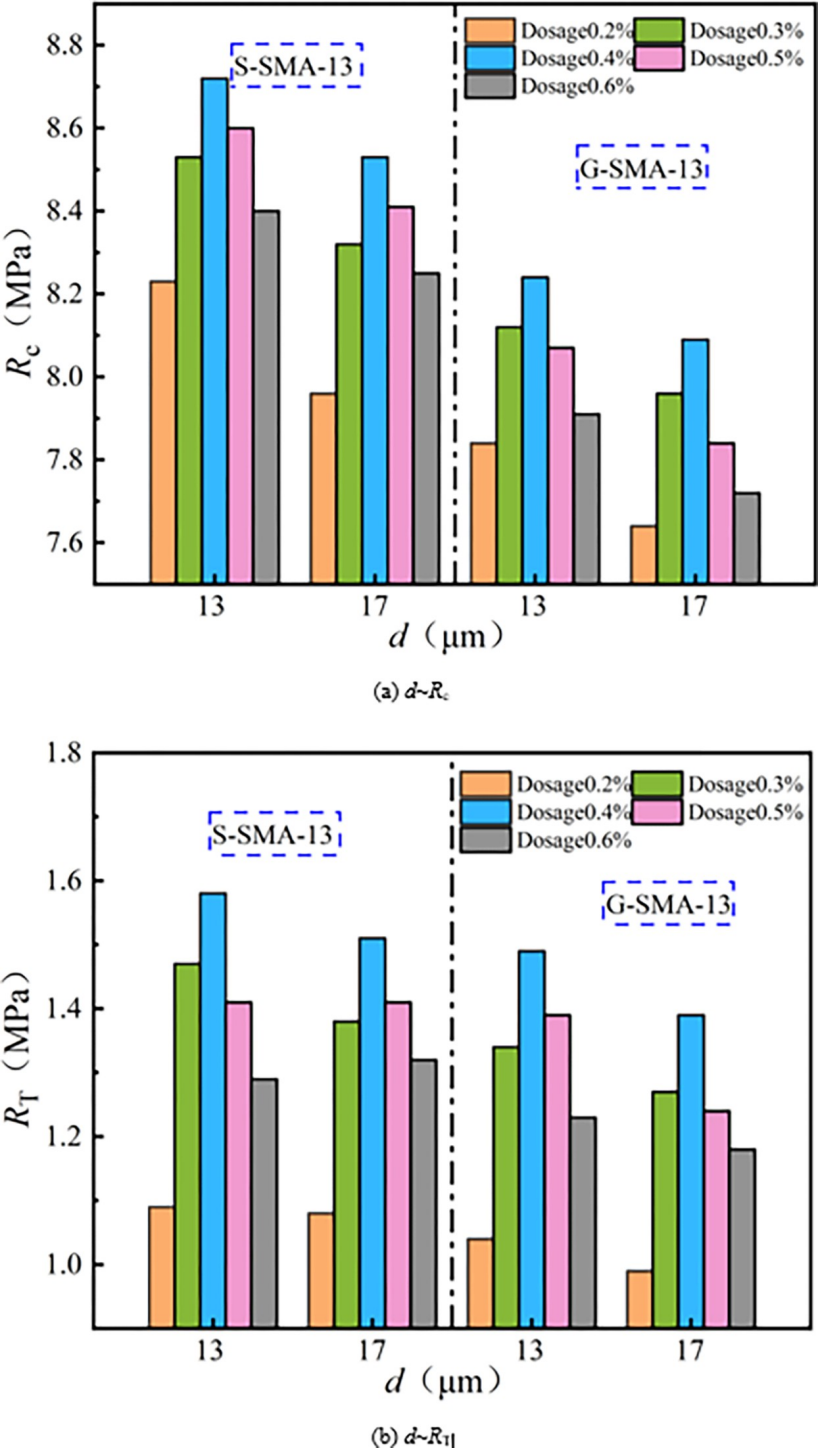
Influence of fiber diameter on mechanical properties.

The effect of basalt fiber diameter on compressive strength (*R*_*c*_) and splitting strength (*R*_*T*_) of SMA-13 is illustrated in Fig 5.

As illustrated in Fig 5, the compressive strength and splitting strength of SMA-13 containing basalt fibers with a diameter of 13 μm are both greater than those of SMA-13 containing basalt fibers with a diameter of 17 μm. The average compressive strength and splitting strength of SMA-13 mixed with 13 μm diameter basalt fibers are 2.4% and 4.5% higher, respectively, than those mixed with 17 μm diameter basalt fibers. The smaller diameter fibers are more easily dispersed uniformly within the SMA-13 mixture, possess a larger specific surface area, and bond with more asphalt, thereby enhancing the cohesive strength of the mix. The incorporation of finer basalt fibers aids in the distribution of the three phases within the SMA-13 mixture, effectively disperses the load carried by the mixture, and consequently improves its mechanical properties.

(2) High-temperature stability

**Fig 6:**
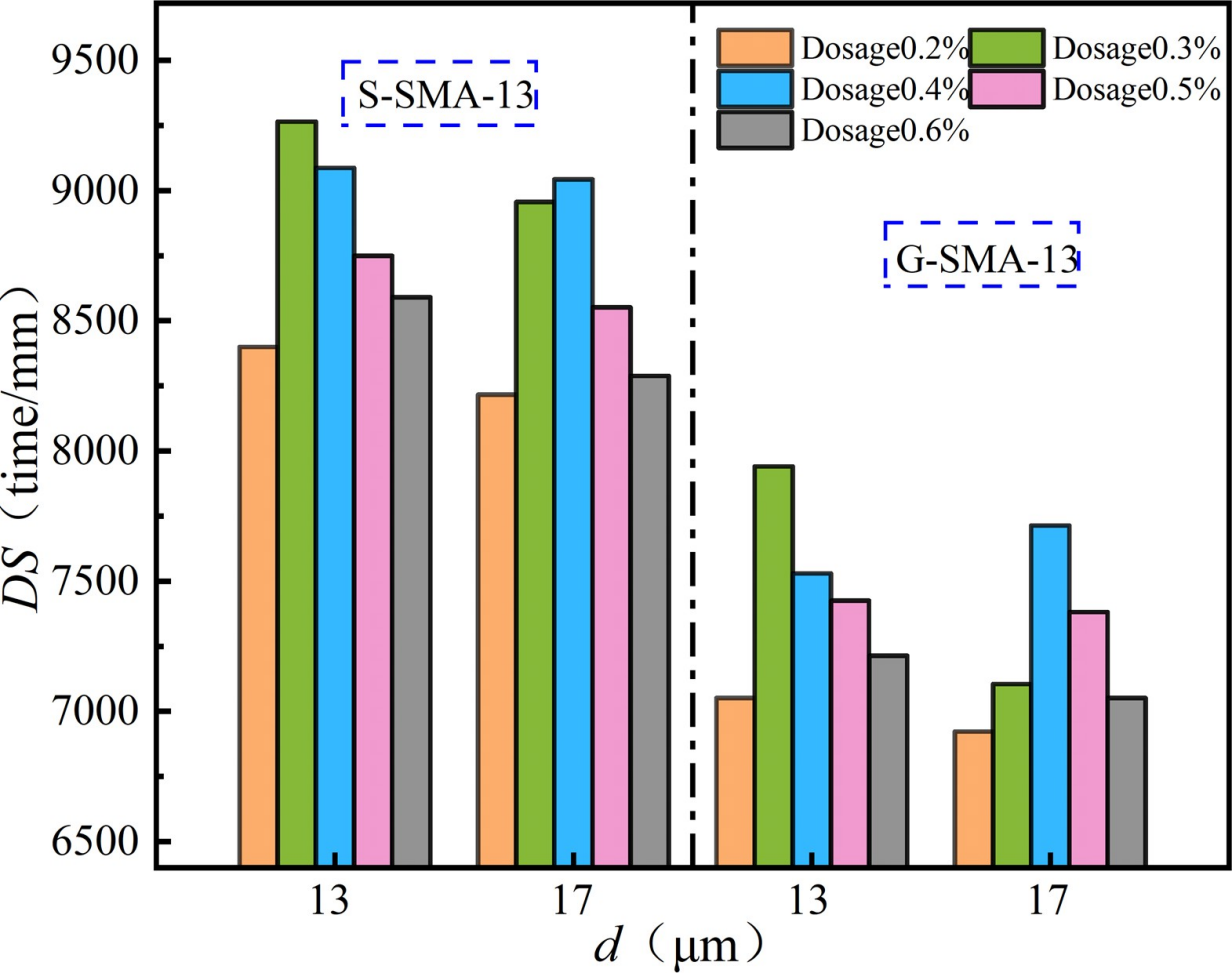
Influence of fiber diameter on high-temperature stability.

The effect of basalt fiber diameter on high-temperature stability of SMA-13 is illustrated in Fig 6.

It can be observed from Fig 6 that the dynamic stability of SMA-13 containing basalt fibers with a diameter of 13 μm is greater than that of SMA-13 containing basalt fibers with a diameter of 17 μm. Specifically, the dynamic stability of SMA-13 mixed with 13 μm basalt fibers is 2.6% higher than that of SMA-13 mixed with 17 μm basalt fibers. The finer diameter of the basalt fibers results in a greater quantity being present at the same content. A larger amount of basalt fiber adsorbs asphalt in the SMA-13 mixture, leading to the formation of structural asphalt and a reduction in the amount of free asphalt in the mixture. The network skeleton structure created by the fibers restricts the flow of free asphalt, thereby enhancing the high-temperature stability of the mixture.

(3) Low-temperature cracking resistance

**Fig 7:**
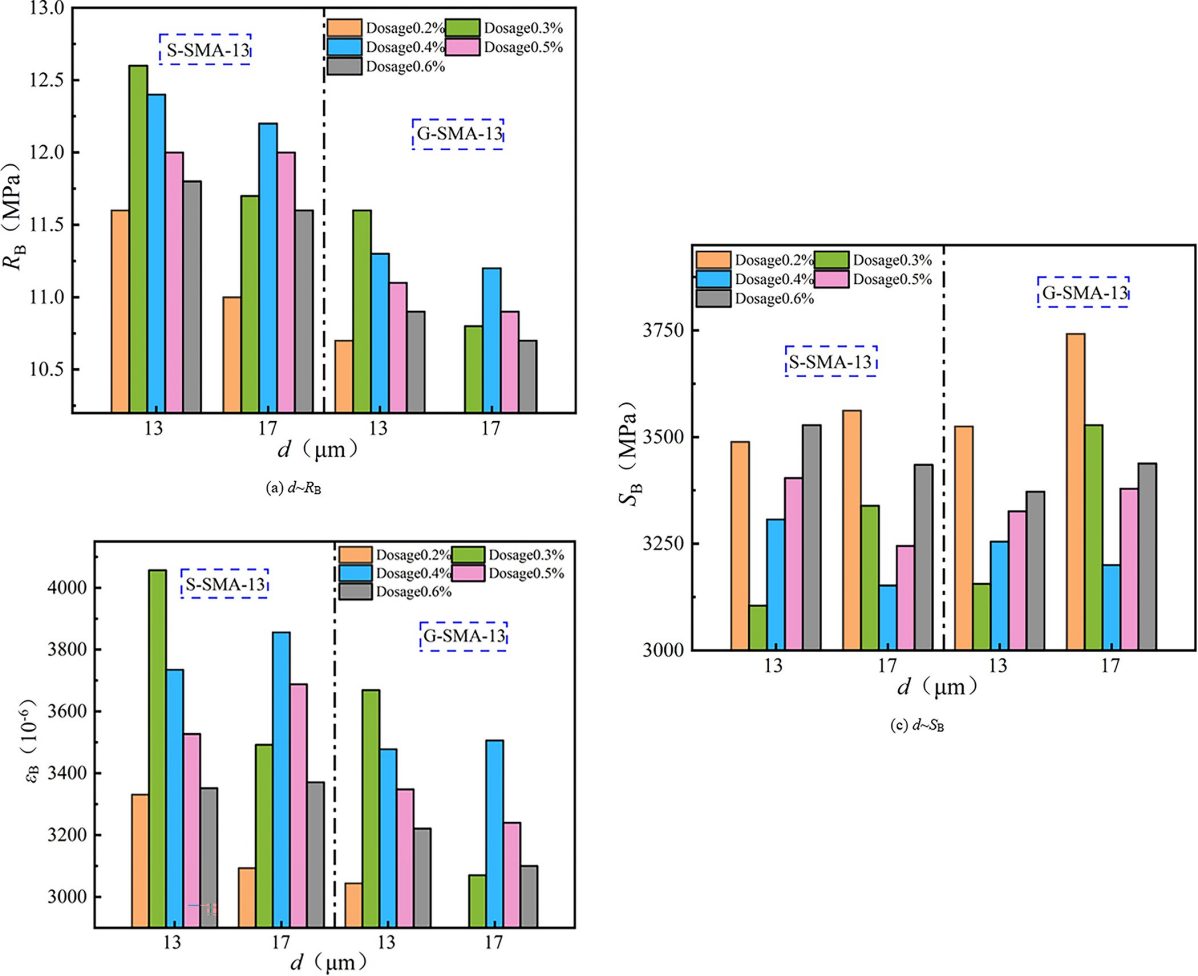
Effect of fiber diameter on low-temperature cracking resistance.

The effect of basalt fiber diameter on low-temperature cracking resistance of SMA-13 is illustrated in Fig 7.

As illustrated in Fig 7, the low-temperature cracking resistance of SMA-13 mixed with basalt fiber of 13 μm diameter is superior to that of SMA-13 mixed with basalt fiber of 17 μm diameter. The bending tensile strength, bending tensile strain, and bending stiffness modulus of SMA-13 containing 13 μm basalt fiber are, on average, 3.3% higher, 4.8% higher, and 1.6% lower, respectively, compared to those of SMA-13 containing 17 μm basalt fiber. Finer basalt fibers are more effective in enhancing the SMA-13 mixture’s ability to resist cracking and improve its toughness. Basalt fiber possesses a high elastic modulus and tensile strength. When the SMA-13 mixture experiences cracking under load, the fibers can absorb a portion of the energy from the damaging load, thereby enhancing the mixture’s low-temperature cracking resistance.

(4) Water stability

**Fig 8:**
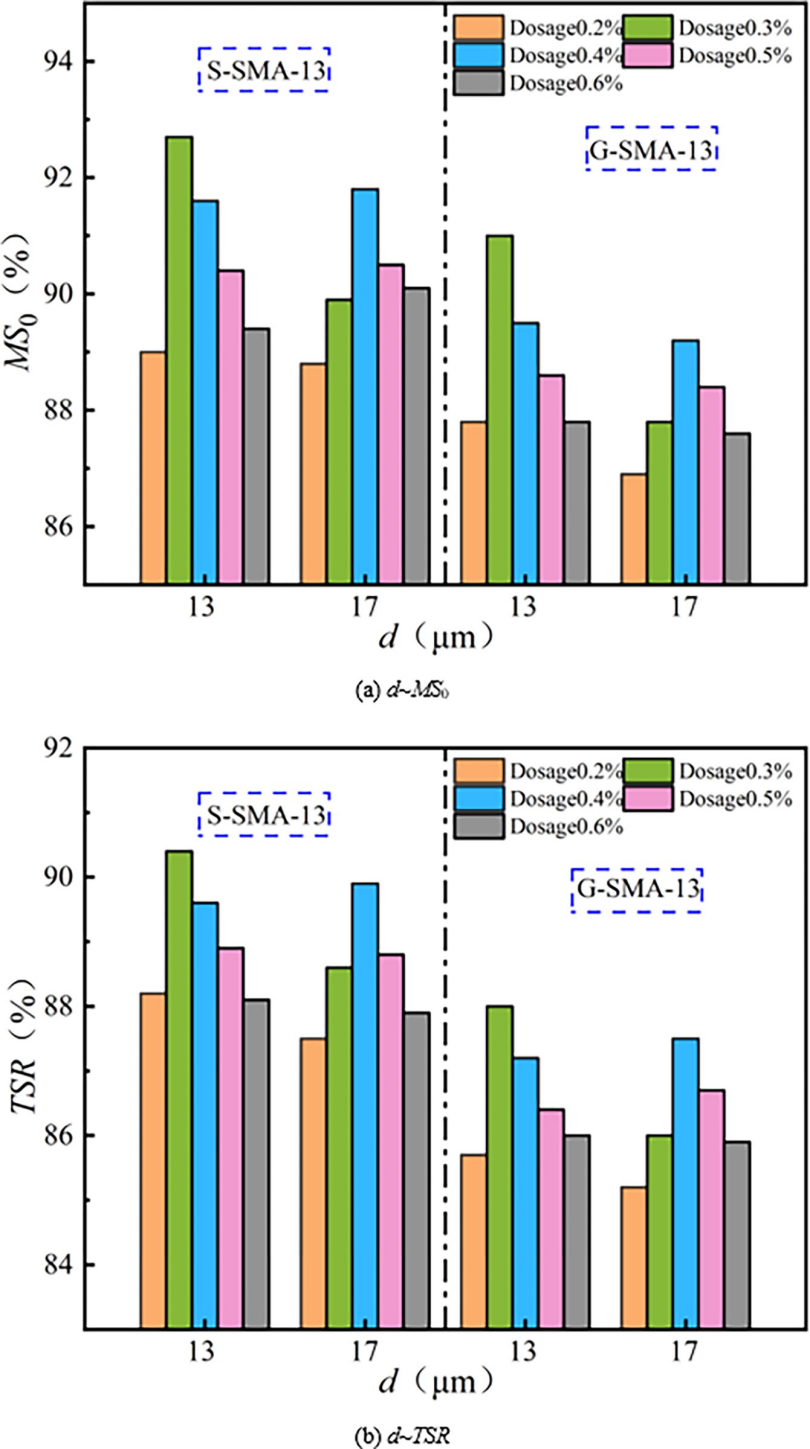
Influence rule of diameter on water stability.

The effect of basalt fiber diameter on water stability of SMA-13 is illustrated in Fig 8.

As illustrated in Fig 8, the water stability performance of SMA-13 with a basalt fiber diameter of 13 μm is superior to that of SMA-13 with a basalt fiber diameter of 17 μm. The residual stability and freeze-thaw splitting strength of the 13 μm basalt fiber SMA-13 mixture was 0.8% higher than those of the 17 μm basalt fiber SMA-13 mixture. The water stability performance of the basalt fiber SMA-13 mixture varies slightly with different fiber diameters because the diameter of the fiber influences its bonding capacity with asphalt. Water stability primarily depends on the bonding strength between asphalt and mineral aggregates, as well as the compactness of the mixture. Although finer fibers exhibit improved water stability due to their larger specific surface area, their impact on the bond strength of the mixture is limited.

### Effect of fiber length

(1) Mechanical property

**Fig 9:**
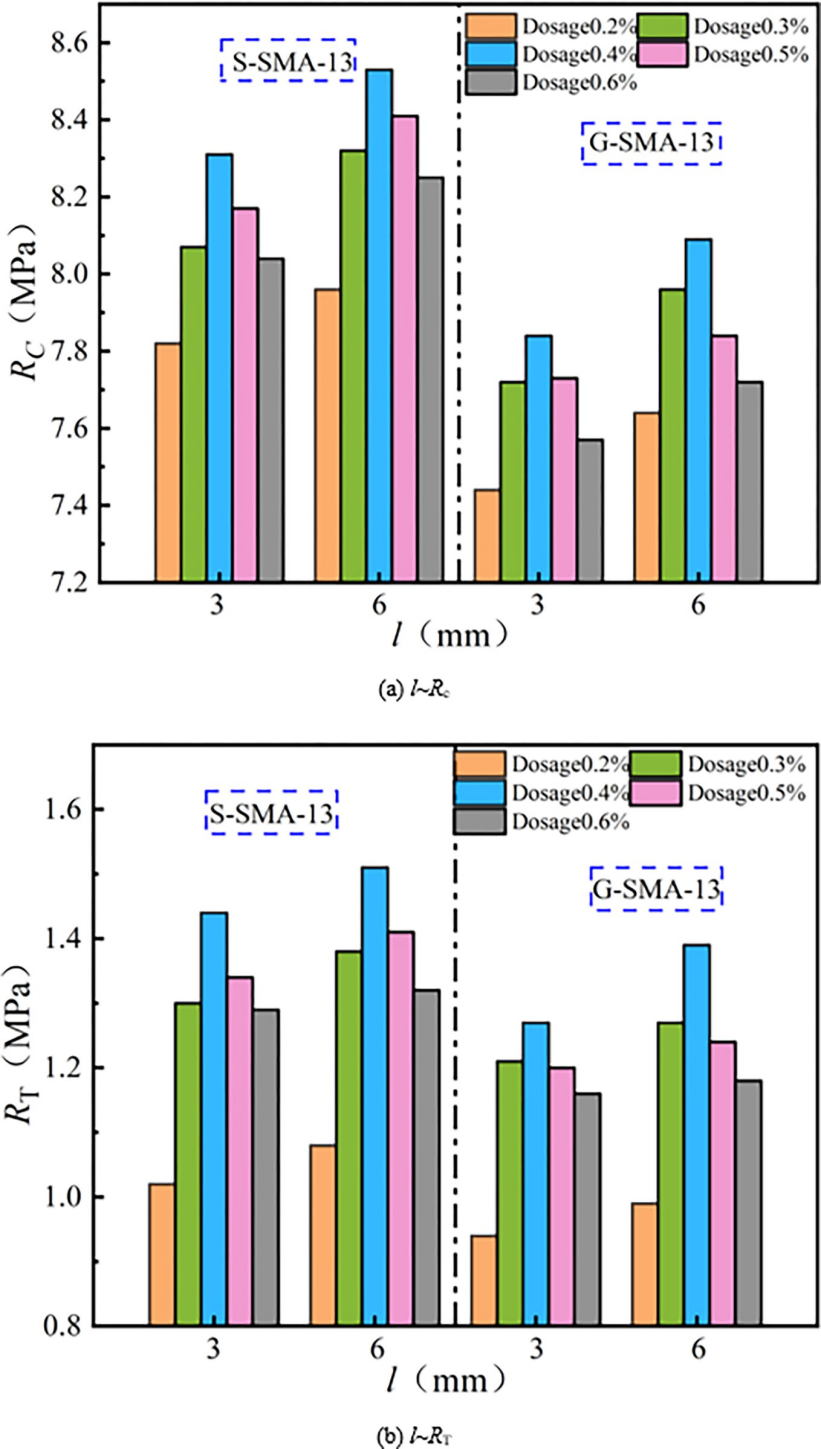
Influence of fiber length on mechanical properties.

The effect of basalt fiber length on the mechanical properties of SMA-13 is illustrated in Fig 9.

It can be observed from Fig 9 that the compressive strength and splitting strength of SMA-13 containing basalt fibers with a length of 6 mm are greater than those of SMA-13 containing basalt fibers with a length of 3 mm. The SMA-13 mixture with 6 mm basalt fibers exhibits an average compressive strength that is 2.6% higher and a splitting strength that is 4.8% higher than that of the mixture with 3 mm basalt fibers. As the length of the basalt fibers increases, the reinforcement effect on the SMA-13 mixture becomes more pronounced. When the longer fibers are incorporated into the mixture, they interlace to form a spatial network structure, which enhances the cohesion of the mixture and improves its mechanical properties.

(2) High-temperature stability

**Fig 10:**
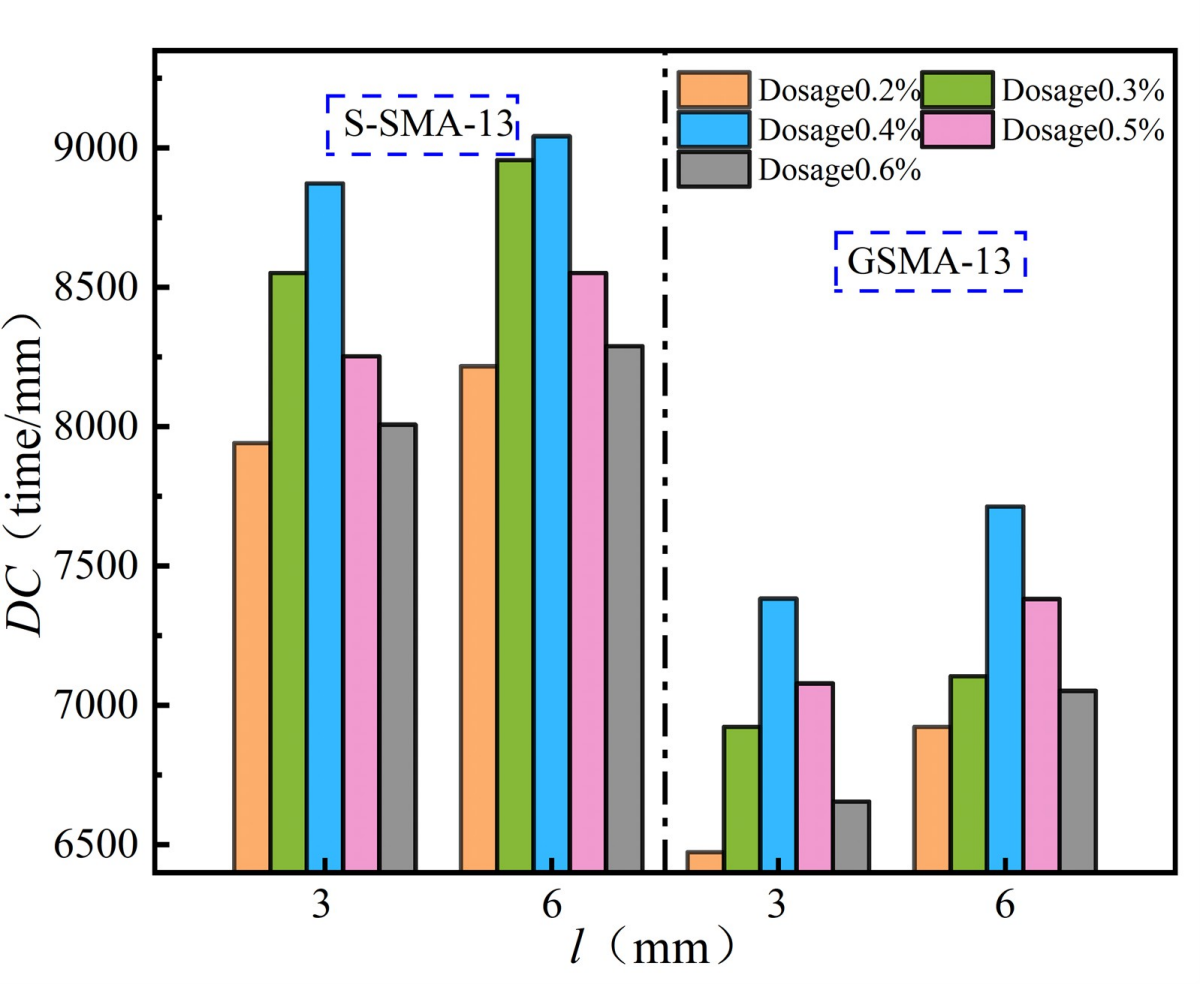
Influence rule of length on high-temperature stability.

The effect of fiber length on high-temperature stability of SMA-13 is shown in Fig 10.

As illustrated in Fig 10, the dynamic stability of SMA-13 with a basalt fiber length of 6 mm is greater than that of SMA-13 with a basalt fiber length of 3 mm. Additionally, the dynamic stability of SMA-13 mixed with 13 μm diameter basalt fiber is 4.1% higher than that of SMA-13 mixed with 17 μm diameter basalt fiber. A longer basalt fiber length increases the likelihood of forming a network structure within the SMA-13 mixture, which restricts lateral displacement under high-temperature conditions and enhances high-temperature stability. Conversely, when the fiber length is short and the interconnections are not sufficiently reinforced, the high-temperature stability of the mixture is compromised.

(3) Low-temperature cracking resistance

**Fig 11:**
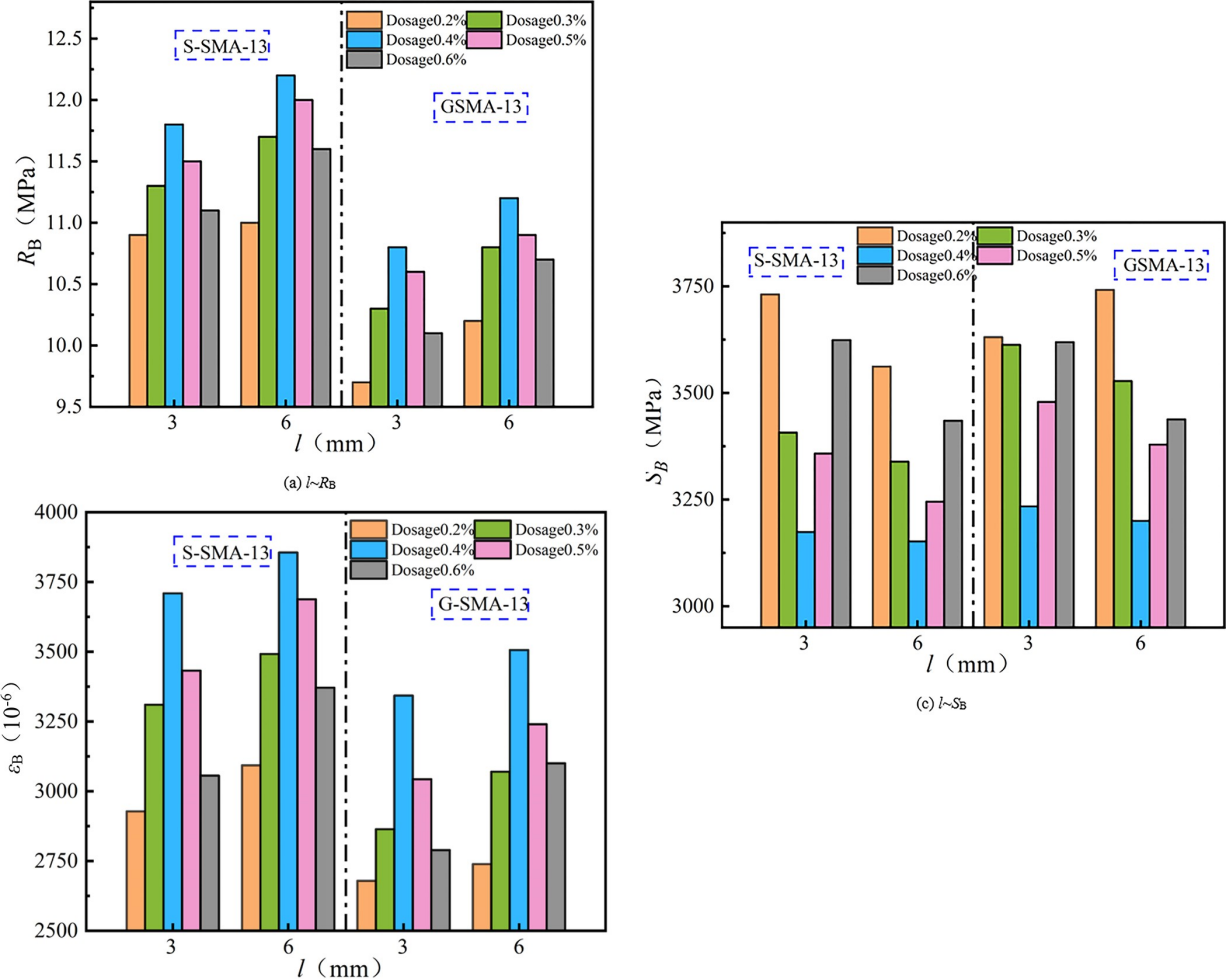
Effect of fiber length on low-temperature cracking resistance.

The effect of fiber length on low-temperature cracking resistance is illustrated in Fig 11.

As illustrated in Fig 11, the low-temperature cracking resistance of SMA-13 with a basalt fiber length of 6 mm is superior to that of SMA-13 with a basalt fiber length of 3 mm. Specifically, the SMA-13 mixture containing 6 mm basalt fibers exhibits a 3.9% increase in flexural tensile strength, a 6.4% increase in flexural tensile strain, and an average decrease of 2.4% in bending stiffness modulus compared to the mixture with 3 mm fibers. Due to its exceptional flexibility, the longer fiber effectively prevents crack propagation by bridging, absorbs more deformation energy, and slows down the propagation of cracks during the initial stages of microcrack formation, thereby enhancing the low-temperature crack resistance of the mixture.

(4) Water stability

**Fig 12:**
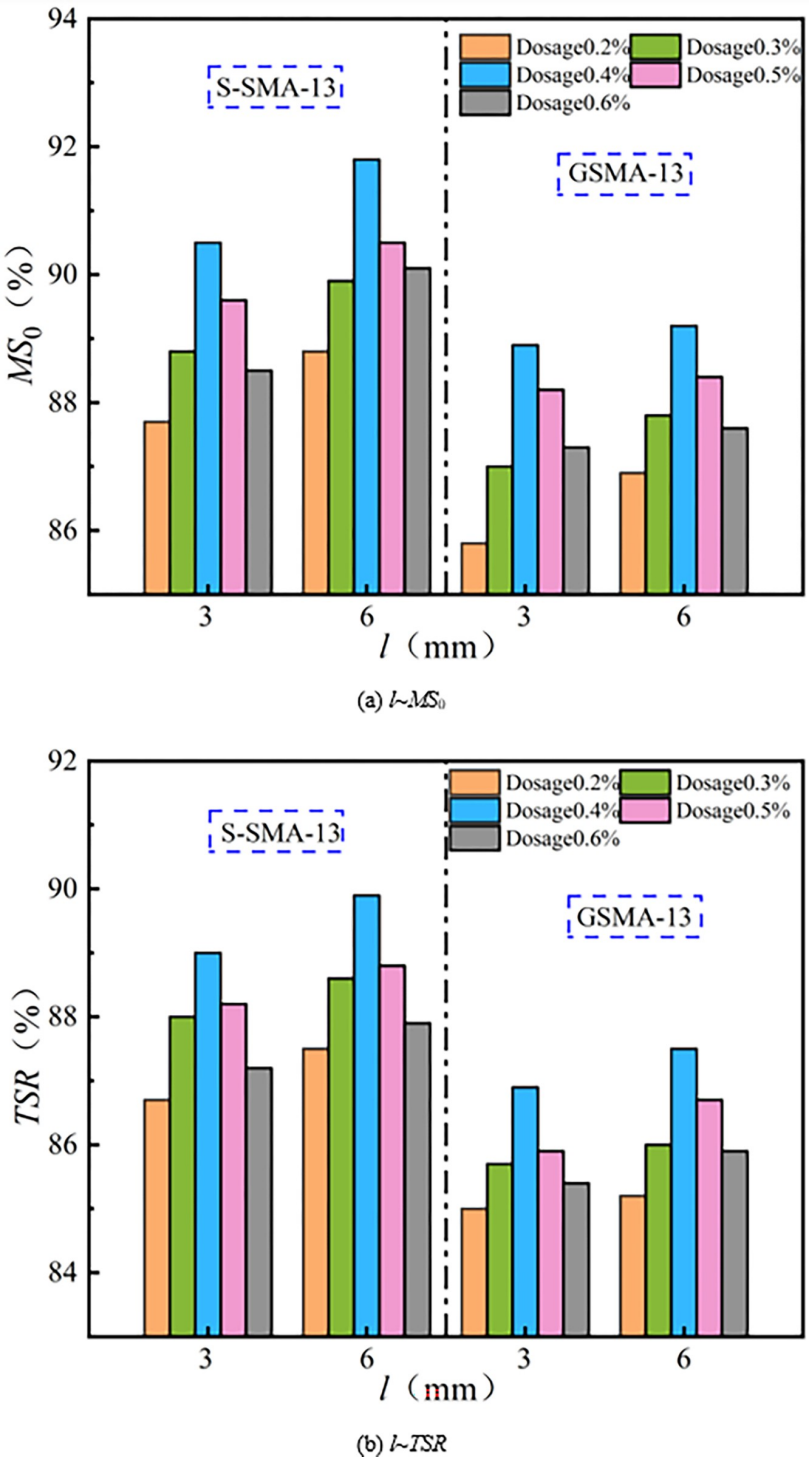
Influence of fiber length on water stability.

The effect of basalt fiber length on the water stability of SMA-13 is illustrated in Fig 12.

It can be observed from Fig 12 that the water stability performance of SMA-13 with a basalt fiber length of 6 mm is superior to that of SMA-13 with a basalt fiber length of 3 mm. The residual stability of the 6 mm basalt fiber SMA-13 mixture is 1.0% higher than that of the 3 mm basalt fiber SMA-13 mixture, and the freeze-thaw splitting strength is, on average, 0.7% higher. The longer fibers can absorb freer leachate than the shorter fibers, forming an asphalt film on the aggregate surface that prevents water intrusion into the mixture, thereby enhancing the water stability of the SMA-13 mixture. The water stability of the mixture is primarily dependent on the bond between the asphalt and the aggregate; thus, the improvement in water stability with the longer fibers is only marginally greater than that of the SMA-13 mixture with shorter fibers.

### Effect of fiber content

(1) Mechanical property

**Fig 13:**
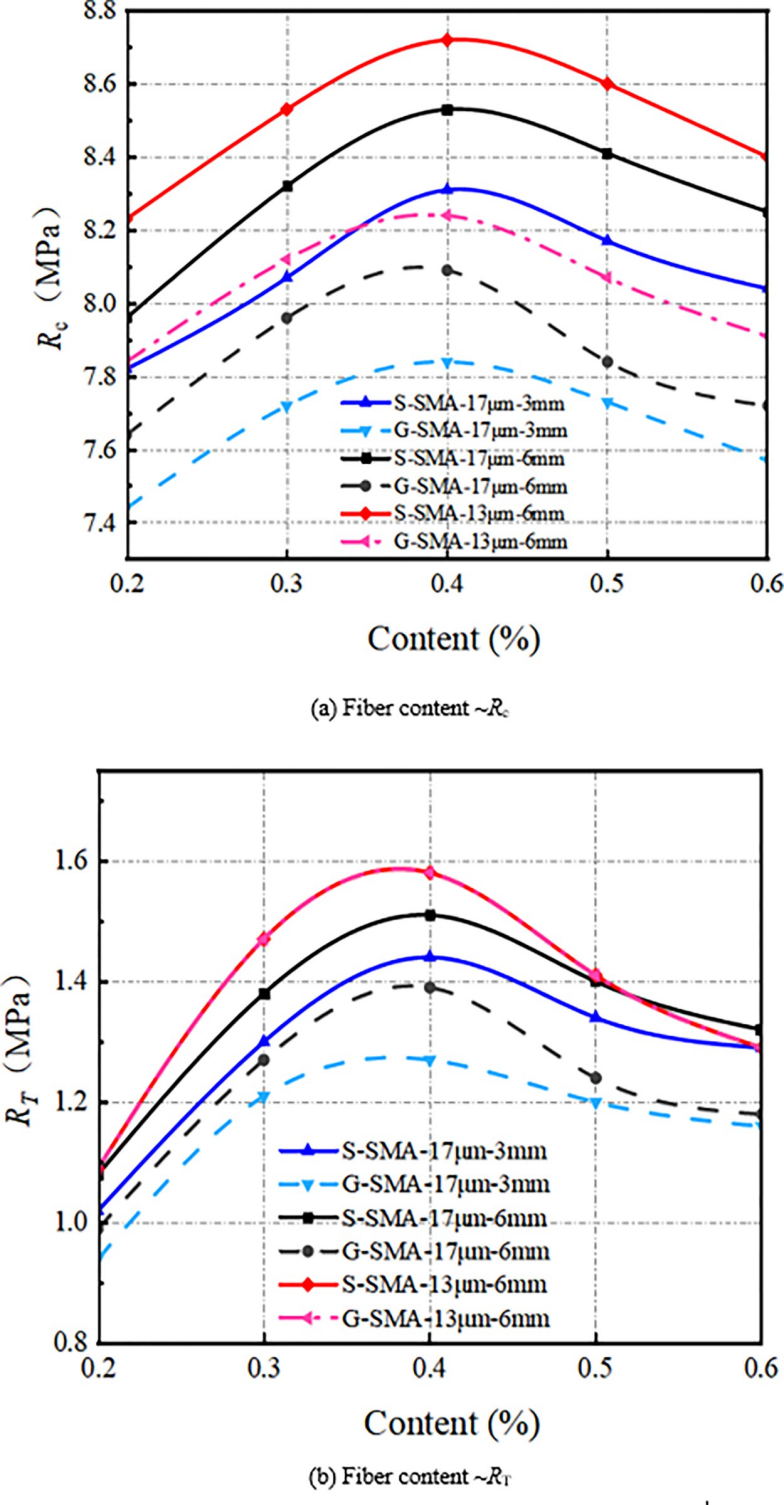
Influence of fiber content on mechanical properties.

The effect of basalt fiber content on the mechanical properties of SMA-13 is illustrated in Fig 13.

As illustrated in Fig 13, the compressive strength and splitting strength of basalt fiber SMA-13 initially increase and then decrease with increasing fiber content, peaking at a content level of 0.4%. This behavior can be attributed to the incorporation of basalt fiber into the asphalt mixture, which effectively disperses load stress, reduces stress concentration, and adsorbs a significant amount of asphalt, thereby forming a boundary layer of asphalt with a certain thickness. The structural asphalt within this boundary layer coats the fibers on the surface of the aggregates, enhancing the cohesion between the aggregates and, consequently, improving the mechanical properties of the mixture. However, as the fiber content continues to rise, the fibers can no longer adsorb additional asphalt or form more structural asphalt, resulting in a decline in the mechanical properties of the mixture.

(2) High-temperature stability

**Fig 14:**
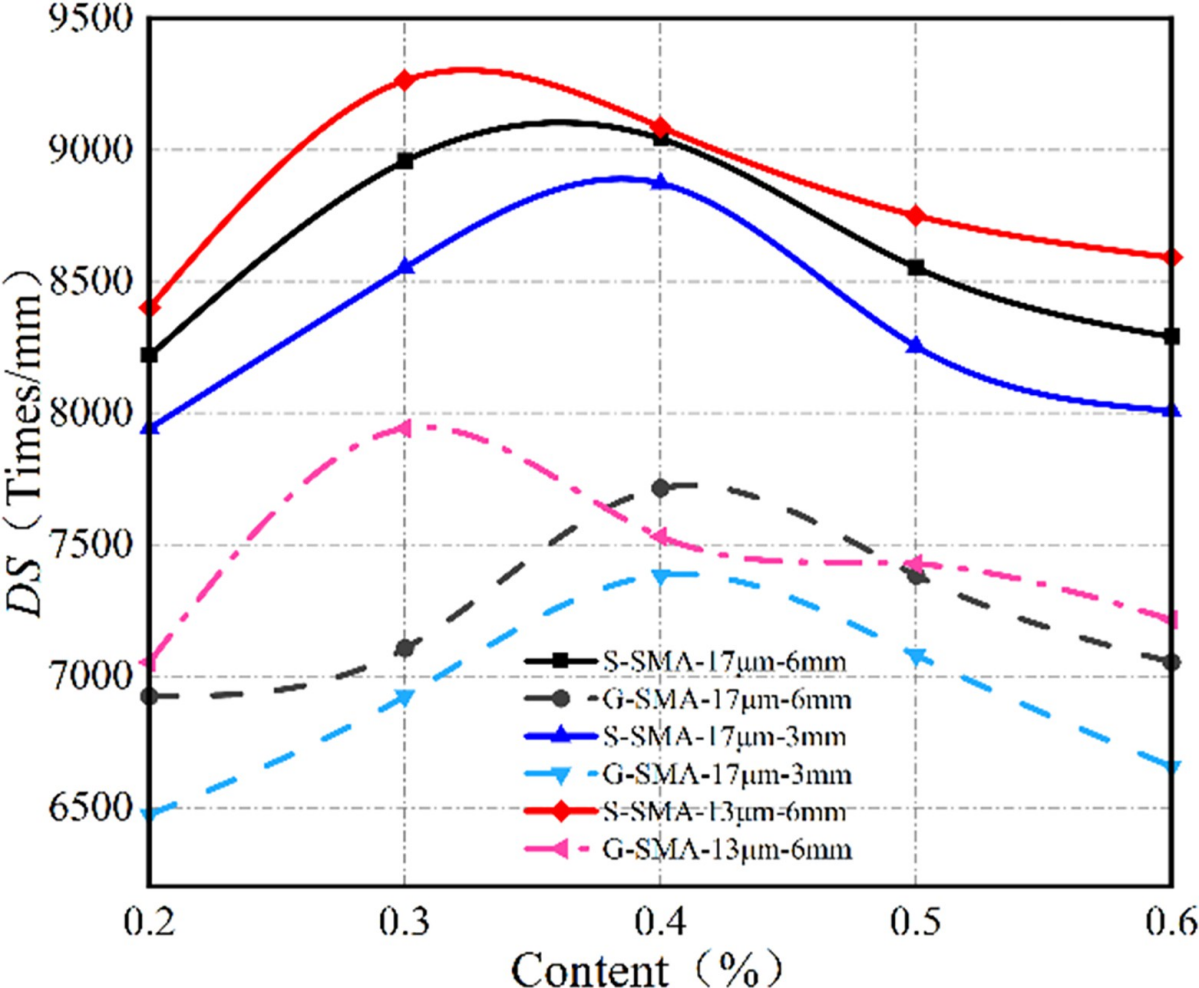
Effect of fiber content on high-temperature stability.

The effect of basalt fiber content on the high-temperature stability of SMA-13 is illustrated in Fig 14.

As illustrated in Fig 14, the dynamic stability of basalt fiber SMA-13 exhibits a downward parabolic relationship with increasing fiber content. The high-temperature stability of S-SMA-13 and G-SMA-13, when mixed with BF-17 μm-6 mm and BF-17 μm-3 mm, reaches its peak at a fiber content of 0.4%. Conversely, the high-temperature stability of S-SMA-13 and G-SMA-13 mixed with BF-13 μm-6 mm is optimal at a fiber content of 0.3%. There exists an optimal content range of basalt fiber that enhances the high-temperature stability of SMA-13. At lower contents, the fibers are evenly dispersed throughout the mixture, forming a crisscross network structure that impedes the flow of free asphalt under high-temperature conditions, thereby improving high-temperature stability. However, as the content continues to increase, the dispersion and uniformity of the fibers within the mixture diminish. This reduction leads to a weakened effect of the fibers on the high-temperature stability of the asphalt mixture, ultimately resulting in decreased dynamic stability.

(3) Low-temperature cracking resistance

**Fig 15:**
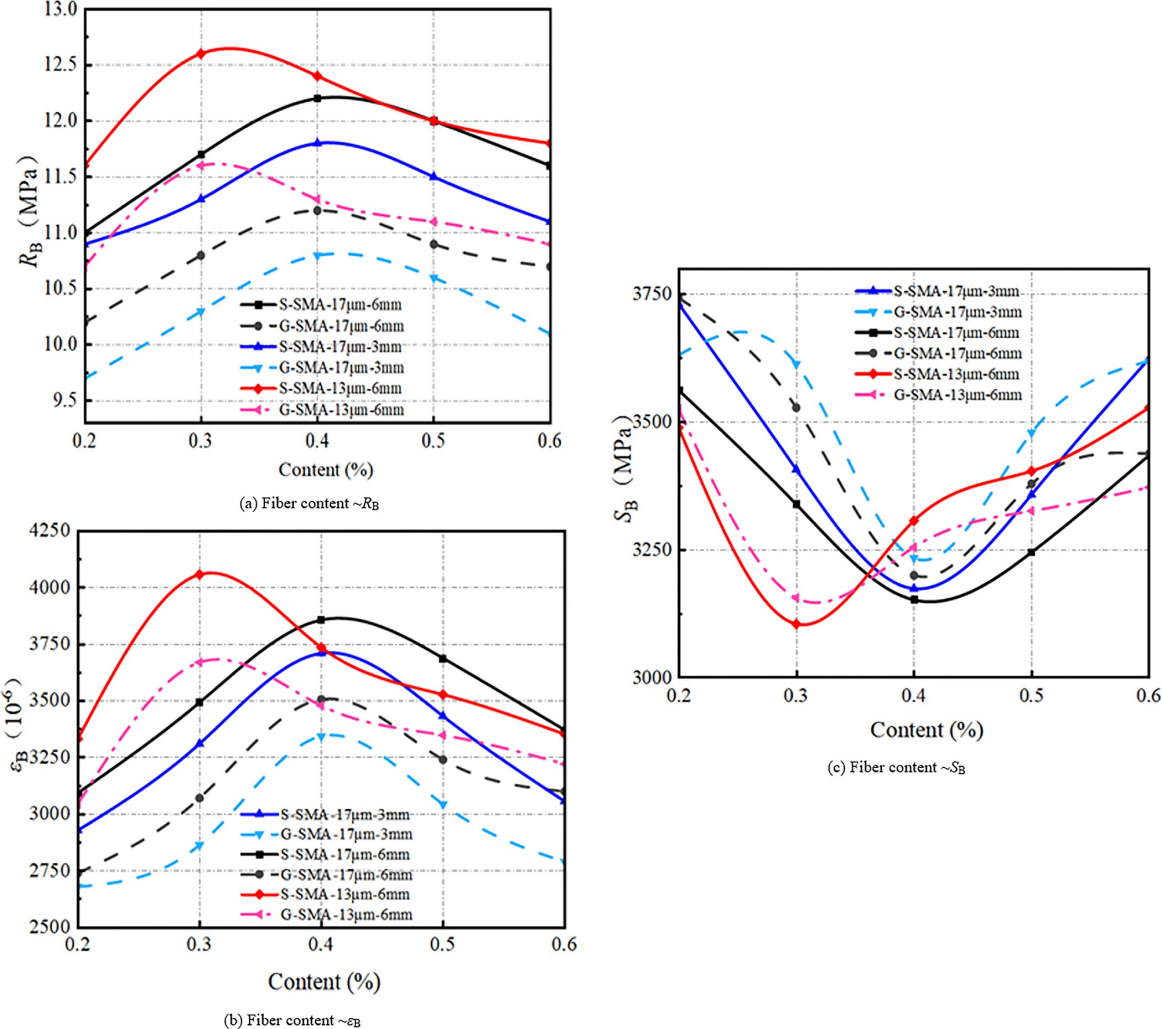
Effect of fiber content on crack resistance at low-temperature.

The effect of basalt fiber content on the low-temperature cracking resistance of SMA-13 is illustrated in Fig 15.

It can be observed from Fig 15 that the flexural tensile strength and flexural tensile strain of basalt fiber-reinforced SMA-13 initially increase and then decrease with increasing fiber content. Conversely, the flexural stiffness modulus first decreases and then increases as fiber content rises. The low-temperature crack resistance of S-SMA-13 and G-SMA-13 mixed with BF-17 μm-6 mm and BF-17 μm-3 mm is optimal at a fiber content of 0.4%. In contrast, the low-temperature crack resistance of S-SMA-13 and G-SMA-13 mixed with BF-13 μm-6 mm is best at a fiber content of 0.3%. Basalt fiber mitigates crack development in the mixture through its bridging action and enhances the toughness of the SMA-13 mixture due to its high elastic modulus and tensile strength, thereby improving the low-temperature crack resistance of the mixture. However, when the fiber content is excessively high, the basalt fiber does not receive sufficient asphalt for effective encapsulation and bonding, leading to fiber aggregation. This aggregation creates areas that are prone to crack initiation and propagation under low-temperatures or loads, ultimately diminishing the effectiveness of the basalt fiber in enhancing the low-temperature crack resistance of the mixture.

(4) Water stability

**Fig 16:**
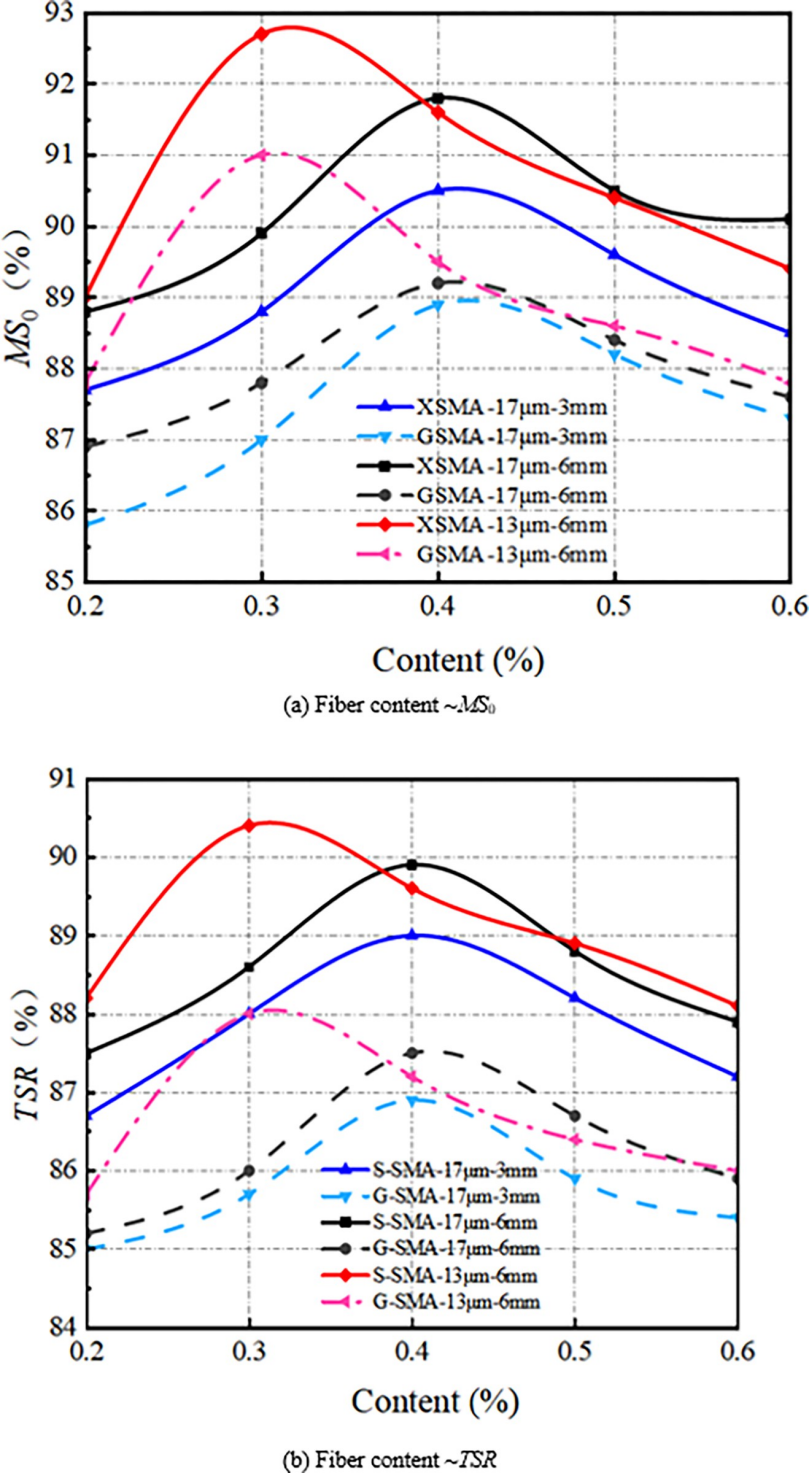
Effect of fiber content on water stability.

The effect of basalt fiber content on the water stability of SMA-13 is illustrated in Fig 16.

It can be observed from Fig 16 that the residual stability and freeze-thaw splitting strength of the basalt fiber SMA-13 mixture exhibit a downward-opening parabolic relationship with increasing fiber residue. The water stability performance of S-SMA-13 and G-SMA-13 mixed with BF-17 μm-6 mm and BF-17 μm-3 mm is optimal at a content of 0.4%. In contrast, the water stability performance of S-SMA-13 and G-SMA-13 mixed with BF-13 μm-6 mm is best at a content of 0.3%. Basalt fiber possesses a large specific surface area, which allows it to adsorb free asphalt and form a thick structural asphalt film between the asphalt and aggregate. This process enhances the adhesion between the asphalt and aggregate, thereby improving the water stability of the mixture. However, when the content of basalt fiber is excessively high, its dispersion within the mixture’s spatial structure deteriorates. An overabundance of basalt fiber can lead to the disruption of the mixture’s microstructure, increased porosity, and the creation of pathways for water intrusion, ultimately weakening the enhancement effect on the mixture’s water stability.

In summary, when the basalt fiber with diameter of 17 μm and length of 6 mm and diameter of 17 μm and length of 3 mm are added at 0.4%, the circuit performance of SMA-13 is the best. When the basalt fiber diameter is 13 μm and length is 6 mm, the circuit performance of SMA-13 is the best when the content is 0.3%.

## Weight analysis of the influence of basalt fiber on asphalt-aggregate ratio and pavement performance

Random Forest is an ensemble learning model that consists of multiple decision trees. The results from each decision tree contribute to the final classification and regression outcomes. This model is particularly effective for influence ranking and weight analysis of multi-factor variables. The algorithm enhances the accuracy of the proposed model by integrating the predictions of several decision trees.

### Influence weight analysis of asphalt-aggregate ratio

By calculating the optimal amount of asphalt, the ideal ratio of the SMA-13 mixture was determined based on the diameter, length, and content of basalt fiber, as presented in Table 11.

**Table 11:** Asphalt-aggregate ratios under different fibers.

Content (%)	Diameter (μm)	Length (mm)	S-SMA-asphalt-aggregate ratio	G-SMA-asphalt-aggregate ratio
0.2	13	6	5.80	5.90
0.2	17	6	5.79	5.89
0.2	17	3	5.78	5.88
0.3	13	6	5.85	5.94
0.3	17	6	5.83	5.92
0.3	17	3	5.83	5.90
0.4	13	6	5.88	5.96
0.4	17	6	5.86	5.95
0.4	17	3	5.85	5.93
0.5	13	6	5.9	6.00
0.5	17	6	5.89	5.99
0.5	17	3	5.88	5.96
0.6	13	6	5.93	6.05
0.6	17	6	5.91	6.03
0.6	17	3	5.9	6.01

**Fig 17:**
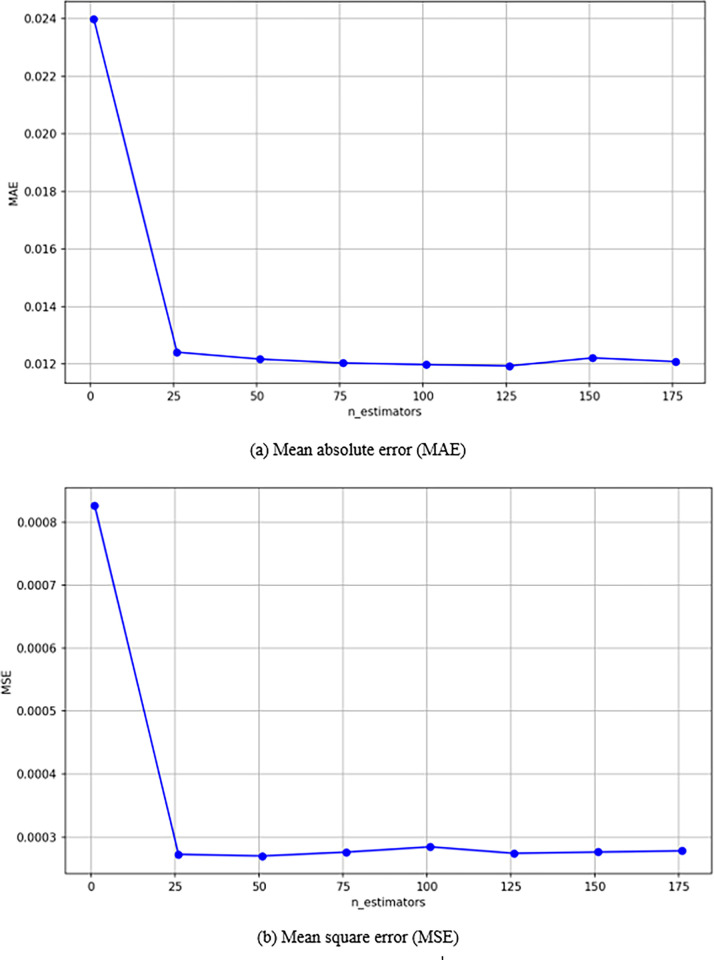
Learning curve.

In practical applications, the training set typically comprises 2/3 to 4/5 of the total data. For this study, the sample data was divided using an 8:2 ratio, with 80% allocated to the training set and 20% to the test set. The characteristics selected for analysis included diameter, length, and content, while the asphalt-aggregate ratio was designated as the target variable. The test set was created with a total label value and a test size of 0.2, using a random state of 42. To optimize the model, a learning curve was employed to adjust the number of trees. The parameter n_estimators define the number of decision trees to be considered, with values ranging from 1 to 200 in increments of 25. These values represent the number of iterations during the random forest training process. The dataset was divided into five parts, and the cross-validation method was utilized to conduct iterative training and assess the average absolute error for each random forest regressor. The results of the cross-validation are presented in Fig 17.

As illustrated in Fig 17, the random forest model demonstrates that both errors and fluctuations gradually decrease as the number of trees increases. When the number of trees exceeds 25, the amplitude of fluctuations diminishes, and the error remains stable, not exceeding 0.001. In the case of the earlier breakpoint fluctuation, with the number of trees set at 125, more accurate prediction results can be achieved while maintaining a certain level of stability.

The weight ranking of the influencing factors obtained from the trained model is presented in Tables 12 and 13.

**Table 12:** S-SMA- Feature importance ranking table.

Sort	Influencing factor	Weighted value
1	Content	0.9233
2	Diameter	0.0447
3	Length	0.0320

**Table 13:** G-SMA- Feature importance ranking table.

Sort	Influencing factor	Weighted value
1	Content	0.9199
2	Diameter	0.0466
3	Length	0.0335

The following results can be obtained from Tables 12 and 13:

(1) The fiber content is the most significant characteristic among all the factors analyzed. In the S-SMA mixture, its weight accounts for 0.960, while the diameter (second most significant) and length (third most significant) have lesser influences, with weights of 0.024 and 0.016, respectively. In the G-SMA mixture, the weights are 0.9199, 0.0466, and 0.0335, respectively.

(2) The fiber content significantly influences the asphalt-aggregate ratio of the mixture. From the standpoint of the mixture’s bonding performance, the addition of fiber alters its bonding characteristics. To preserve optimal bonding performance, it may be necessary to increase the asphalt content and adjust the asphalt-aggregate ratio accordingly.

(3) The characteristics of fiber length and diameter are relatively unimportant, likely because their variation does not significantly affect the overall performance of the fiber in the mix or the asphalt demand. Additionally, a uniform distribution of fibers in the mix and appropriate content adjustments further mitigate this effect.

### Influence weight analysis of pavement performance

The division of the dataset into training and test sets plays an essential role in traditional machine learning, particularly in the context of random forests. As previously mentioned, the sample data is divided in an 80:20 ratio, with 80% allocated to the training set and 20% to the test set.

Using diameter, length, and content as characteristic data, and SMA-13 routing performance index as label data, a random forest model was established. The parameters were adjusted through random matching and exhaustive matching. The range for each type of hyperparameter was defined and combined. The number of decision trees was selected from 50 to 200, while the maximum depth of the decision trees was chosen from 10 to 40, with an increment of 50. RandomizedSearchCV was employed for random ordering, and the optimal combination was output. To ensure a globally optimal combination of hyperparameters, GridSearchCV was utilized to explore each match, allowing for the selection of a superior final hyperparameter value. By comparing the model results for each combination of hyperparameter values, the optimal hyperparameter set was identified, and the model was trained. Additionally, the influence of variable importance was analyzed.

(1) Mechanical property

Length, diameter, and content were considered independent variables, while compressive strength and splitting strength were treated as dependent variables for the random forest modeling. The results are presented in Table 14.

**Table 14:** Weight chart of factors influencing mechanical properties.

Influencing factor	The weight value of S-SMA-*R*_c_	The weight value of S-SMA-*R*_T_	The weight value of G-SMA-*R*_c_	The weight value of G-SMA-*R*_T_
Length	0.175	0.030	0.047	0.019
Diameter	0.356	0.054	0.645	0.190
Content	0.469	0.916	0.308	0.791

It can be observed from Table 14 that fiber content and diameter significantly influence the mechanical properties of SMA-13, whereas fiber length has a comparatively minor effect. Fiber content and diameter directly impact the overall distribution and network structure of the fibers within the mixture. Adjusting fiber content and diameter is more easily controllable during the processing and construction phases, facilitating the achievement of a stable ratio and uniform distribution throughout the production process. Conversely, an increase in fiber length can complicate the processing and construction of the mixture, leading to issues such as uneven mixing and challenges in ensuring proper fiber distribution during construction. These complications may hinder the potential for significant improvements in the strength properties associated with increased fiber length. Consequently, the effect of fiber length on the compressive strength and splitting strength of the mixture is relatively minimal.

(2) High-temperature stability

Length, diameter, and content were considered independent variables, while dynamic stability was treated as the dependent variable for random forest modeling. The results are presented in Table 15.

**Table 15:** Weight chart of factors affecting high-temperature stability.

Influencing factor	The weight value of S-SMA-*DS*	The weight value of G-SMA-*DS*
Length	0.112	0.191
Diameter	0.282	0.209
Content	0.606	0.600

It can be observed from Table 15 that fiber content and diameter significantly influence the high-temperature stability of SMA-13, whereas fiber length has a lesser impact. Both fiber content and diameter are crucial in forming an effective fiber network structure and in enhancing the bonding with the matrix. The improvement in dynamic stability associated with fiber length primarily manifests in crack control and the enhancement of toughness. However, dynamic stability is more significantly affected by the structure of the fiber network and the bonding between the fiber and the matrix, resulting in a relatively minor influence of fiber length on the dynamic stability of the mixture.

(3) Low-temperature crack resistance

Length, diameter, and content were considered independent variables, while flexural tensile strength, flexural stiffness modulus, and flexural tensile strain were treated as dependent variables for random forest modeling. The results are presented in Table 17.

It can be observed from Table 16 that the fiber content and diameter significantly influence the low-temperature cracking resistance of SMA-13, whereas the fiber length has a comparatively minor effect. Although altering the fiber length may enhance the material’s toughness, the fiber content and diameter have a more direct impact on fiber distribution within the matrix and the formation of the network structure. A well-formed network structure can more effectively distribute stress, thereby increasing both the flexural tensile strength and the bending stiffness modulus. Fiber content and diameter are easier to control and can be evenly distributed, while longer fibers in the mixture may result in uneven distribution or handling difficulties, which can adversely affect the overall performance of the material. Consequently, fiber content and diameter typically lead to more significant improvements in material performance.

**Table 16:** Weight chart of factors influencing low-temperature crack resistance.

Influencing factor	The weight value of S-SMA-*R*_B_	The weight value of S-SMA-*ε*_B_	The weight value of S-SMA-*S*_B_	The weight value of G-SMA-*R*_B_	The weight value of G-SMA-*ε*_B_	The weight value of G-SMA-*S*_B_
Length	0.124	0.126	0.150	0.180	0.113	0.118
Diameter	0.353	0.182	0.104	0.367	0.289	0.230
Content	0.522	0.692	0.746	0.453	0.598	0.652

(4) Water stability

Length, diameter, and content were considered independent variables, while residual stability and the freeze-thaw splitting strength ratio were treated as dependent variables for random forest modeling. The results are presented in Table 17.

**Table 17:** Weight chart of influencing factors of water stability.

Influencing factor	The weight value of S-SMA-*MS*_0_	The weight value of S-SMA-*TSR*	The weight value of G-SMA-*MS*_0_	The weight value of G-SMA-*TSR*
Length	0.160	0.113	0.079	0.075
Diameter	0.149	0.271	0.252	0.196
Content	0.690	0.616	0.669	0.729

It can be observed from Table 17 that both fiber content and diameter significantly influence the water stability of SMA-13, whereas fiber length has a minimal effect. The effect of fiber content on the fiber network structure and bond strength is more direct, leading to a substantial enhancement in the overall performance of the mixture. Although longer fibers can contribute additional toughness, their role in establishing an effective fiber network structure is generally less significant compared to fiber content and diameter. Additionally, long fibers may be unevenly distributed within the mix, potentially compromising uniformity and, consequently, the overall performance of the mixture.

## Conclusions

Taking SMA-13 as an example, the combined effects of basalt fiber geometrical characteristics (fiber diameter, length, and content) on the pavement performance of asphalt mixtures are investigated. Moreover, a random forest algorithm is adopted to conduct a weight analysis of fiber characteristics and its correlation with pavement performance. The main conclusions are as follows:

(1) The influence of basalt fiber diameter, length, and content on the optimal asphalt-aggregate ratio of SMA-13 is examined. The results indicate that the optimal asphalt-aggregate ratio of SMA-13 is not influenced by the diameter or length of the basalt fiber. However, the optimal asphalt-aggregate ratio of SMA-13 increases linearly with an increase in basalt fiber content. The degree of influence of basalt fiber on the optimal asphalt-aggregate ratio is ranked as follows: 13 μm-6 mm > 17 μm-6 mm > 17 μm-3 mm.

(2) The influence of basalt fiber diameter, length, and content on the pavement performance of SMA-13 was studied. The results indicate that the pavement performance of SMA-13 is not significantly affected by the diameter or length of the basalt fiber. However, as the content of basalt fiber increases, the pavement performance of SMA-13 initially improves and then declines, suggesting the existence of an optimal content range. The circuit performance of SMA-13 is optimal with basalt fiber diameters of 17 μm and 13 μm at contents of 0.4% and 0.3%, respectively.

(3) The fiber content is the most significant characteristic among all the factors analyzed. In the S-SMA mixture, its weight accounts for 0.960, while the diameter (second most significant) and length (third most significant) have lesser influences, with weights of 0.024 and 0.016, respectively. In the G-SMA mixture, the weights are 0.9199, 0.0466, and 0.0335, respectively.

(4) The fiber content significantly influences the asphalt-aggregate ratio of the mixture. From the perspective of bond performance, the addition of fiber alters the bond characteristics of the mixture. To ensure optimal bond performance, it may be necessary to increase the asphalt content and adjust the asphalt-aggregate ratio accordingly.

(5) The characteristics of fiber length and diameter are relatively unimportant, likely because their variation does not significantly affect the overall properties of the fiber in the mixture. Additionally, uniform distribution of the fibers in the mix and appropriate content adjustments help mitigate this effect.

## Supporting information

S1 File. Data This file includes all the test data of the asphalt mixtures. (DOCX)
