# Supplementary material for: Combined effects of basalt fiber geometrical characteristics on pavement performance of asphalt mixtures
Source: PLoS One. 2025 Jan 31;20(1):e0316173. doi: 10.1371/journal.pone.0316173 (PMC11785331; doi:10.1371/journal.pone.0316173)
Supplement: S1 File. Data — (DOCX) [file pone.0316173.s001.docx]

**Best oil stone ratio of basalt fiber SMA-13**

| Gradation type | Basalt fiber type | The following dosage (%) SMA-13 optimum oil stone ratio (%) | | | | |
| --- | --- | --- | --- | --- | --- | --- |
|  |  | 0.2 | 0.3 | 0.4 | 0.5 | 0.6 |
| S-SMA-13 | BF-17 μm-3 mm | 5.78 | 5.83 | 5.85 | 5.88 | 5.90 |
|  | BF-17 μm-6 mm | 5.79 | 5.83 | 5.86 | 5.89 | 5.91 |
|  | BF-13 μm-6 mm | 5.80 | 5.85 | 5.88 | 5.90 | 5.93 |
| G-SMA-13 | BF-17 μm-3 mm | 5.88 | 5.90 | 5.93 | 5.96 | 6.01 |
|  | BF-17 μm-6 mm | 5.89 | 5.92 | 5.95 | 5.99 | 6.03 |
|  | BF-13 μm-6 mm | 5.90 | 5.94 | 5.96 | 6.00 | 6.05 |

**Mechanical and road performance test results of basalt fiber SMA-13 (BF-17 μm-3 mm)**

| Gradation type | Dosage (%) | *R*_c_ (MPa) | *R*_T_ (MPa) | *DS*  (times/mm) | *R*_B_  (MPa) | *ε*_B_  (10^-6^) | *S*_B_ (MPa) | *MS*_0_ (%) | *TSR* (%) |
| --- | --- | --- | --- | --- | --- | --- | --- | --- | --- |
| X-SMA-13 | 0.2 | 7.8 | 1.02 | 7941 | 10.9 | 2928 | 3731 | 87.7 | 86.7 |
|  | 0.3 | 8.1 | 1.30 | 8552 | 11.3 | 3310 | 3407 | 88.8 | 88.0 |
|  | 0.4 | 8.3 | 1.44 | 8873 | 11.8 | 3709 | 3174 | 90.5 | 89.0 |
|  | 0.5 | 8.2 | 1.34 | 8253 | 11.5 | 3432 | 3358 | 89.6 | 88.2 |
|  | 0.6 | 8.0 | 1.29 | 8008 | 11.1 | 3056 | 3624 | 88.5 | 87.2 |
| G-SMA-13 | 0.2 | 7.4 | 0.94 | 6473 | 9.7 | 2679 | 3631 | 85.8 | 85.0 |
|  | 0.3 | 7.7 | 1.21 | 6923 | 10.3 | 2864 | 3613 | 87.0 | 85.7 |
|  | 0.4 | 7.8 | 1.27 | 7383 | 10.8 | 3343 | 3234 | 88.9 | 86.9 |
|  | 0.5 | 7.7 | 1.20 | 7079 | 10.6 | 3043 | 3479 | 88.2 | 85.9 |
|  | 0.6 | 7.6 | 1.16 | 6655 | 10.1 | 2789 | 3619 | 87.3 | 85.4 |

**Mechanical and road performance test results of basalt fiber SMA-13 (BF-17 μm-6 mm)**

| Gradation type | Dosage (%) | *R*_c_ (MPa) | *R*_T_ (MPa) | *DS*  (times/mm) | *R*_B_  (MPa) | *ε*_B_  (10^-6^) | *S*_B_ (MPa) | *MS*_0_ (%) | *TSR* (%) |
| --- | --- | --- | --- | --- | --- | --- | --- | --- | --- |
| S-SMA-13 | 0.2 | 8.0 | 1.08 | 8217 | 11.0 | 3093 | 3562 | 88.8 | 87.5 |
|  | 0.3 | 8.3 | 1.38 | 8957 | 11.7 | 3492 | 3339 | 89.9 | 88.6 |
|  | 0.4 | 8.5 | 1.51 | 9043 | 12.2 | 3856 | 3152 | 91.8 | 89.9 |
|  | 0.5 | 8.4 | 1.40 | 8552 | 12.0 | 3688 | 3245 | 90.5 | 88.8 |
|  | 0.6 | 8.3 | 1.32 | 8289 | 11.6 | 3371 | 3435 | 90.1 | 87.9 |
| G-SMA-13 | 0.2 | 7.6 | 0.99 | 6923 | 10.2 | 2739 | 3742 | 86.9 | 85.2 |
|  | 0.3 | 8.0 | 1.27 | 7105 | 10.8 | 3070 | 3528 | 87.8 | 86.0 |
|  | 0.4 | 8.1 | 1.39 | 7714 | 11.2 | 3506 | 3200 | 89.2 | 87.5 |
|  | 0.5 | 7.8 | 1.24 | 7382 | 10.9 | 3240 | 3379 | 88.4 | 86.7 |
|  | 0.6 | 7.7 | 1.18 | 7052 | 10.7 | 3100 | 3438 | 87.6 | 85.9 |

**Mechanical and road performance test results of basalt fiber SMA-13 (BF-13 μm-6 mm)**

| Gradation type | Dosage (%) | *R*_c_ (MPa) | *R*_T_ (MPa) | *DS*  (times/mm) | *R*_B_  (MPa) | *ε*_B_  (10^-6^) | *S*_B_ (MPa) | *MS*_0_ (%) | *TSR* (%) |
| --- | --- | --- | --- | --- | --- | --- | --- | --- | --- |
| S-SMA-13 | 0.2 | 8.23 | 1.09 | 8400 | 11.6 | 3331 | 3489 | 89.0 | 88.2 |
|  | 0.3 | 8.53 | 1.47 | 9265 | 12.6 | 4057 | 3105 | 92.7 | 90.4 |
|  | 0.4 | 8.72 | 1.58 | 9087 | 12.4 | 3735 | 3307 | 91.6 | 89.6 |
|  | 0.5 | 8.60 | 1.41 | 8750 | 12.0 | 3527 | 3404 | 90.4 | 88.9 |
|  | 0.6 | 8.40 | 1.29 | 8591 | 11.8 | 3352 | 3528 | 89.4 | 88.1 |
| G-SMA-13 | 0.2 | 7.84 | 1.04 | 7052 | 10.7 | 3044 | 3525 | 87.8 | 85.7 |
|  | 0.3 | 8.12 | 1.34 | 7941 | 11.6 | 3669 | 3156 | 91.0 | 88.0 |
|  | 0.4 | 8.24 | 1.49 | 7530 | 11.3 | 3478 | 3255 | 89.5 | 87.2 |
|  | 0.5 | 8.07 | 1.39 | 7426 | 11.1 | 3348 | 3326 | 88.6 | 86.4 |
|  | 0.6 | 7.91 | 1.23 | 7214 | 10.9 | 3221 | 3372 | 87.8 | 86.0 |

**The ratio of oil to stone under different fibers**

| Content | Diameter | Length | S-SMA-Oil-stone ratio | G-SMA-Oil-stone ratio |
| --- | --- | --- | --- | --- |
| 0.2 | 13 | 6 | 5.8 | 5.90 |
| 0.2 | 17 | 6 | 5.79 | 5.89 |
| 0.2 | 17 | 3 | 5.78 | 5.88 |
| 0.3 | 13 | 6 | 5.85 | 5.94 |
| 0.3 | 17 | 6 | 5.83 | 5.92 |
| 0.3 | 17 | 3 | 5.83 | 5.90 |
| 0.4 | 13 | 6 | 5.88 | 5.96 |
| 0.4 | 17 | 6 | 5.86 | 5.95 |
| 0.4 | 17 | 3 | 5.85 | 5.93 |
| 0.5 | 13 | 6 | 5.9 | 6.00 |
| 0.5 | 17 | 6 | 5.89 | 5.99 |
| 0.5 | 17 | 3 | 5.88 | 5.96 |
| 0.6 | 13 | 6 | 5.93 | 6.05 |
| 0.6 | 17 | 6 | 5.91 | 6.03 |
| 0.6 | 17 | 3 | 5.9 | 6.01 |
